# Multi-omics analyses reveal cellular responses to biosynthesis of the non-natural cofactor nicotinamide cytosine dinucleotide

**DOI:** 10.3389/fbioe.2026.1954596

**Published:** 2026-09-29

**Authors:** Xiaojia Guo, Qian Wang, Ting Liu, Yinghan Hu, Xueying Wang, Zongbao K. Zhao

**Affiliations:** 1 MOE Key Laboratory of Bio-Intelligent Manufacturing, School of Bioengineering, Dalian University of Technology, Dalian, China; 2 Laboratory of Biotechnology, Dalian Institute of Chemical Physics, CAS, Dalian, China; 3 SCIEX. China, Shanghai, China

**Keywords:** cellular response, multi-omics, nicotinamide cytosine dinucleotide, non-natural cofactor, selective metabolic regulation

## Abstract

The ubiquitous cofactors nicotinamide adenine dinucleotide (NAD) and its reduced form play pivotal roles in energy metabolism and redox homeostasis. Recently, an NAD analog nicotinamide cytosine dinucleotide (NCD) has emerged for selective energy transfer *in vivo*. However, the biological effects of NCD remain elusive. Here we document cellular responses to NCD biosynthesis by multi-omics analysis of NCD self-sufficient *Escherichia coli* strains. Proteomics analysis revealed that NCD biosynthesis led to disturbance in up to 4% of the proteome, most of which were nonessential proteins or isozymes. By combining omics data and flux analysis, we identified fructose 6-phosphate, cytidine triphosphate, aspartate and glutamate as key branch nodes in the perturbation network. Importantly, we constructed several lines of *E. coli* cells capable of synthesizing NCD at different levels and confirmed that these cells grew similarly to their parental strains in either synthetic minimal or nutrient-rich media. Furthermore, we demonstrated that upregulation of the NCDH/NCD ratio caused little disturbance to those of natural nicotinamide cofactors. Our results assure that biosynthesis of NCD has minimal effects on cell growth and that the redox states of NCD(H) are largely segregated from those of natural redox cofactors, thereby facilitating a broader application of NCD as a non-natural cofactor in chemical biology and synthetic biology.

## 1 Introduction

Cellular energy metabolism and redox homeostasis are fundamental processes in all biological systems. For many redox reactions, the ubiquitous cofactors nicotinamide adenine dinucleotide (NAD) and nicotinamide adenine dinucleotide phosphate (NADP) are involved, and their corresponding reduced forms NADH and NADPH can deliver reducing power for energy transfer or biosynthesis (Kissman et al., 2024; Lee et al., 2019). To optimize the utilization of reducing power toward target compounds, one of the main strategies is to balance energy allocation between the endogenous metabolism and the engineered pathways (Chen R et al., 2022; Ge et al., 2025; Tran et al., 2025). Substantial efforts have been made to enhance product formation via manipulating redox cofactor supply, such as engineering cofactor specificity of enzymes, improving driving forces and redirecting reducing equivalents to the target pathway (Black et al., 2023; Montaño López et al., 2021).

However, NAD(P) participates in global metabolism and the maintenance of redox homeostasis, and perturbation of the supply, allocation and transfer of NAD(P) can cause complex disturbances in cells (Katsyuba et al., 2020; Xiao et al., 2018; Xu et al., 2018). Besides, as universal cofactors, NAD(P) is promiscuously shared by numerous enzymes, and thus the synthesis of each metabolite is intrinsically mediated by a limited yet unpredictable amount of these cofactors (An et al., 2025). To expand the redox cofactor repertoire, non-natural cofactors that mimic the function of natural ones but have different structures have been explored recently (Black et al., 2023; Ji et al., 2011; Lim and Banta, 2025; Reardon, 2024). For instance, nicotinamide mononucleotide (NMN) was used as a noncanonical redox cofactor both *in vitro* and in *Escherichia coli* cells (Aspacio et al., 2024; Liu et al., 2021a; Meng et al., 2023; Paul et al., 2014; Richardson et al., 2020).

Previously, we demonstrated that an NAD analog nicotinamide cytosine dinucleotide (NCD, Figure 1A) could be used as a redox cofactor, and acquired several NCD-preferring enzymes through directed evolution (Guo et al., 2020a; Ji et al., 2011; Liu et al., 2019a). By constructing NCD-linked metabolic circuits, we found that NCD could act as an effective cofactor for pathway-selective energy transfer (Guo et al., 2020a; Wang et al., 2017). To make NCD for *in vivo* applications, we then created NCD synthetase and NCD self-sufficient *E. coli* cells in which the concentration of NCD reached millimolar level without supplementation of any precursors (Wang et al., 2021). However, cellular responses to NCD biosynthesis remain unexplored, although it is essential to rationally engineer NCD-linked metabolism. In general, accumulation of a specific metabolite can lead to substantial changes in terms of cell metabolism and physiology. For example, enhanced production of D-pantothenate and vitamin B6 led to fluctuations in central metabolism (Tian et al., 2024; Wang et al., 2022). For the noncanonical cofactor NMN, its reduced form can cause complicated disturbance *in vivo* and inhibit cell growth (Liu et al., 2021b). While NCD *per se* is a new metabolite and its biosynthesis is clearly linked to nucleotide metabolism (Wang et al., 2021), how cells respond to NCD biosynthesis remains unknown.

**FIGURE 1:**
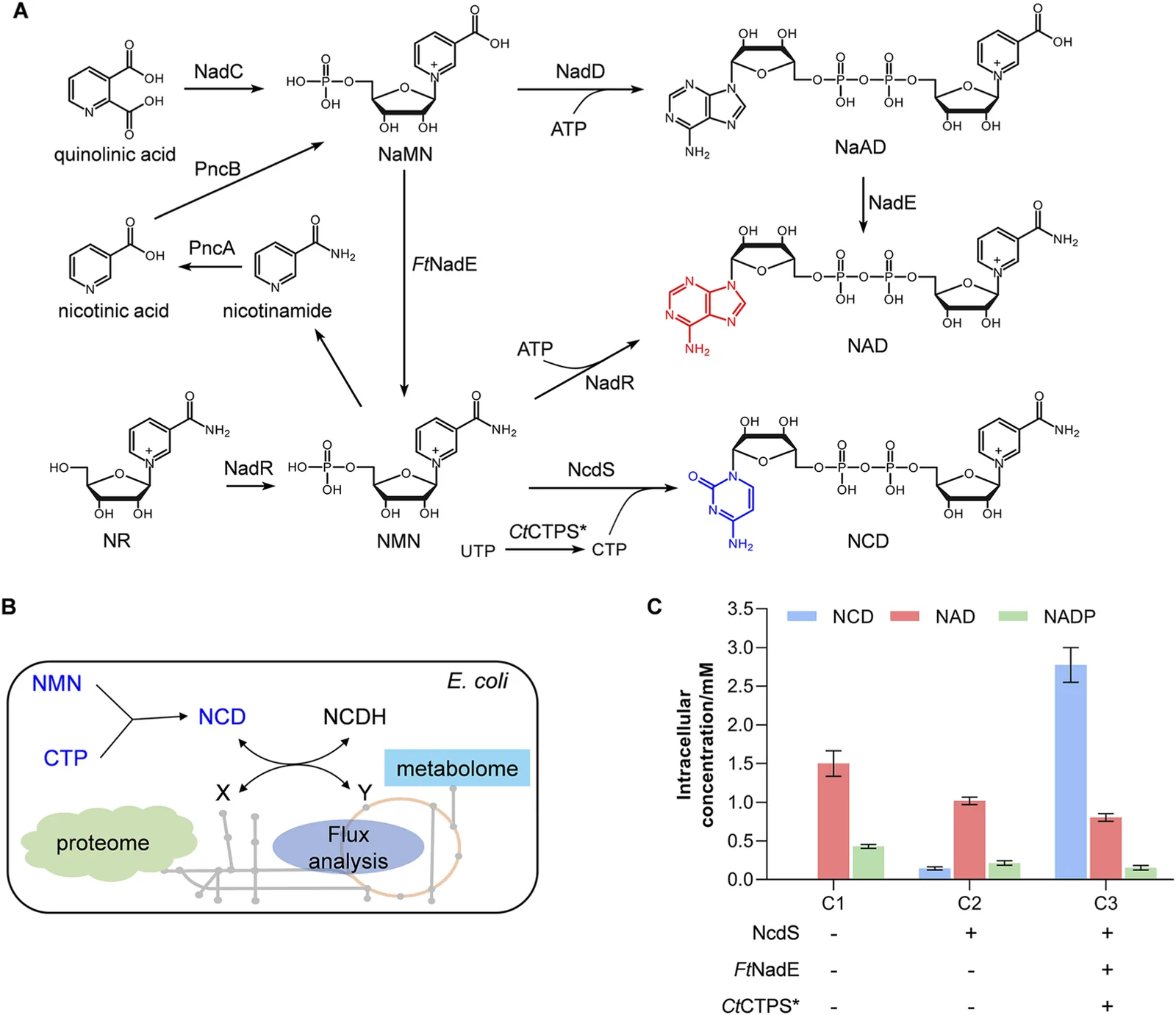
Cofactor synthesis in engineered strains. **(A)** Reactions and enzymes for biosynthesis of NAD and NCD. *Ct*CTPS*, CTP synthetase mutant (D149 E) derived from *Chlamydia trachomatis*; *Ft*NadE, nicotinamide mononucleotide synthetase from *Francisella tularensis*; NadC, quinolinate phosphoribosyltransferase; NadD, nicotinate-nucleotide adenylyltransferase; NadE, NH_3_-dependent NAD synthetase; NadR, NMN adenylyltransferase; NcdS, NCD synthetase; PncA, nicotinamidase; PncB, nicotinate phosphoribosyltransferase; ATP, adenosine triphosphate; CTP, cytidine triphosphate; NaAD, nicotinate adenine dinucleotide; NAD, nicotinamide adenine dinucleotide; NaMN, β-nicotinate D-ribonucleotide; NCD, nicotinamide cytosine dinucleotide; NMN, nicotinamide mononucleotide; NR, Nicotinamide riboside; UTP, uridine triphosphate. **(B)** NCD self-sufficiency and quested omics in this study. X is designated as the electron-donating metabolite, and Y as the electron-accepting metabolite. **(C)** Cofactor concentration in engineered strains. Cells were cultured in 2.5 mL TB medium at 30 °C, 200 rpm for 23 h, and then 1 × 10^10^ cells were collected for cofactor extraction. The data are presented as mean ± SD (n = 3). Source data are provided as a Source Data file.

Here we documented cellular responses at systems level to NCD biosynthesis through integrated analyses of the proteome, metabolome and metabolic flux of NCD self-sufficient strains (Figure 1B). Proteomics analysis revealed that NCD biosynthesis led to disturbance in up to 4% of the proteome, most of which were nonessential proteins or isozymes. Integrated omics analysis and flux balance analysis found that NCD biosynthesis activated the fructose 6-phosphate (F6P) cleavage bypass and had impacts on metabolic nodes of F6P, cytidine triphosphate (CTP), aspartate and glutamate. Finally, we found that there was no cell growth compromise under different nutrient conditions for NCD self-sufficient *E. coli* cells, and that there was little change in terms of the redox states of natural nicotinamide cofactors regardless of the fluctuation of the NCDH/NCD ratio. Our work clarified the biological effects of NCD biosynthesis and confirmed the segregation feature of NCD from natural redox cofactors, which will facilitate a broader application of NCD as a non-natural cofactor in chemical biology and synthetic biology.

## 2 Materials and methods

### 2.1 Strains and media

Strains and plasmids used in this study are shown in Supplementary Table S1. LB medium with 1% tryptone, 0.5% yeast extract and 1% NaCl was used for seed culture. Modified M9 medium was used as the chemostat medium and the feeding medium: 20 g/L glucose, 2 mM MgSO_4_, 0.1 mM CaCl_2_, 6.78 g/L Na_2_HPO_4_, 3 g/L KH_2_PO_4_, 0.5 g/L NaCl, 1 g/L NH_4_Cl, 50 μg/mL carbenicillin, and 1 mM L-arabinose. TB medium with 12 g/L tryptone, 24 g/L yeast extract, 0.4% glycerol, 72 mM K_2_HPO_4_ and 17 mM KH_2_PO_4_ was used for the cultivation of the engineered strains for protein purification and cofactor analysis. M9 medium with MOPS buffer was used for cofactor regulation: 0.4% glucose, 2 mM MgSO_4_, 0.1 mM CaCl_2_, 6.78 g/L Na_2_HPO_4_, 3 g/L KH_2_PO_4_, 0.5 g/L NaCl, 1 g/L NH_4_Cl, 40 mM MOPS (pH 6.5), 50 μg/mL carbenicillin, 50 μg/mL kanamycin, 0.1 mM IPTG and 1 mM L-arabinose.

### 2.2 Chemostat culture

Strains were cultured in LB medium with 50 μg/mL carbenicillin at 37 °C, 200 rpm for 15 h. The culture was centrifuged and the supernatant was removed. Cell pellets were resuspended with modified M9 medium, and then transferred to a 1 L bioreactor (Eppendorf, Hamburg, Germany) at OD_600 nm_ of 0.1 with a working volume of 300 mL. The pH was controlled at 6.5 by automatically adding 2 M NaOH and 2 M HCl solutions. The temperature was maintained at 30° C with the stirrer speed at 400 rpm. When the dissolved oxygen reached a stable level, medium feeding and discharge procedures were started with a dilution rate at 0.05. Samples were taken every 6 h until the glucose concentration and the OD_600 nm_ of the culture reached steady state (Supplementary Figure S1). Cultivation was stopped after 3.5 elution volumes.

Cells were collected by centrifuging at 10,000 *g* for 5 min and washed by PBS three times. A total of 1 × 10^10^ cells were used for metabolomic analysis and 3 × 10^10^ cells were used for proteomics analysis.

### 2.3 Proteomics analysis

Cell samples in triplicates were collected from the chemostat culture. Proteins were extracted with a total protein extraction kit for Gram-negative bacteria (BangFei Bioscience, Beijing, China), and then subjected to SDS-PAGE. Protein concentration was determined by Bradford Kit (Dingguo Changsheng Biotechnology).

Each sample (100 μg) was treated with 5 μL 1 M DTT (Genview, Beijing, China) at 37 °C for 1 h, and then incubated with 20 μL iodoacetamide (Vetec, Darmstadt, Germany) in the absence of light at room temperature for 1 h. After that, samples were centrifuged and washed with 8 M urea and 100 mM Tris-HCl (pH 8.0) twice. Then, 100 μL 0.5 M triethylammonium bicarbonate buffer (Sigma-Aldrich, St. Louis, MO, United States) was used to wash protein samples three times. Proteins were digested by adding trypsin (Promega, Madison, WI, United States) with the ratio of 50:1 (protein: trypsin) at 37 °C for 16 h. Samples of each strain were prepared in triplicates. The digested peptides were labeled with the TMTpro 16pLex Isobaric Label Reagent (Thermo Fisher, Waltham, MA, United States of America) following the instruction. The peptides were labeled as follows: C1-1, 126; C1-2, 127N; C1-3, 127C; C2-1, 128N; C2-2, 128C; C2-3, 129N; C3-1, 132C; C3-2, 131C; C3-3, 134. TMT labeling efficiency was detected by LC-MS/MS.

Samples were first subjected to HPLC grading. Labeled samples were lyophilized and redissolved in phase A (2% acetonitrile, pH 10) (Sigma-Aldrich, St. Louis, MO, United States), and centrifuged at 14,000 g for 20 min after vortex. The supernatant was eluted by a 1,260 infinity II system (Agilent) with an xBridge peptide BEH 130 C18 column (Waters, Milford, MA, United States). The mobile phases were composed of phase A (2% acetonitrile, pH 10) and phase B (98% acetonitrile, pH 10). The gradient varied at the flow rate of 0.7 mL/min. The column was washed by 97% phase A and 3% phase B first, and eluate was collected every 1.5 min from 5 min. Phase A was decreased to 95% at 10 min and continued to decrease to 82% and 66% at 35 min and 45 min, respectively. Phase A was kept at 5% from 53 min to 58 min. The collected samples were lyophilized and redissolved with 5 μL formic acid (Sigma-Aldrich, St. Louis, MO, United States) and then were mixed and separated into different groups. Afterwards, samples from different groups were centrifuged for 10 min and 10 μL of the supernatant was used for injection.

Samples were then applied to LC-MS analysis. Mobile phase A was 0.1% formic acid, and phase B was composed of 0.08% formic acid and 80% acetonitrile. Each sample was analyzed by the Easy nLC/Ultimate 3,000 (Thermo Fisher) system at a flow rate of 600 nL/min with a C18 (3 μm, 100 μm × 20 mm, Thermo Fisher) pre-column and a C18 (1.9 μm, 150 μm × 120 mm, Thermo Fisher) analytical column. Phase A decreased from 94% to 91%, 87%, 74%, 62% and 0% at 2 min, 10 min, 50 min, 70 min and 71 min, respectively. Phase B was kept at 100% from 71 min to 78 min. Mass spectrometry (Orbitrap Fusion, Thermo Fisher) was conducted subsequently with a positive ion spray voltage of 2.3 kV and a negative ion spray voltage of 0.6 kV, and the ion transfer tube temperature was set at 320 °C. MS scan range was between m/z 300–1,400 at an orbitrap resolution of 120 K. For MS/MS, scan range was set at auto normal mode at a resolution of 50 K.

Data were searched by Proteome Discoverer 2.4 against the *E. coli* K12 MG1655 Uniprot database (ID: UP000000625) with the addition of the sequences of overexpressed proteins (Supplementary Table S2). The search parameters were set as follows: enzyme, trypsin; max missed cleavages, 2; fixed modifications, carbamidomethyl (C), TMTpro (N-term), TMTpro (K); variable modifications, oxidation (M), acetyl (protein N-term); peptide mass tolerance, ±10 ppm; fragment mass tolerance, 0.02 Da; peptide confidence, high. The false discovery rate (FDR) was set to ≤0.01 for peptide identification. Differentially expressed proteins were obtained with a fold change threshold of 1.5 and a *p* value of 0.05. The Venn diagram was made by Venny 2.1 (https://bioinfogp.cnb.csic.es/tools/venny/index.html).

### 2.4 Metabolomic analysis

Samples in sextuplicate of each strain were harvested from the chemostat culture and were used for metabolites extraction by pre-cooled methanol: acetonitrile (v:v=1:1) liquid with 30 s of vortex followed by centrifugation at 14,000 rpm for 10 min at −20 °C. A 100 μL aliquot of the supernatant was used for detection, and 10 μL of each sample was mixed for QC analysis.

Samples were then determined by LC/MS (SCIEX ExionLC and SCIEX TripleTOF 6,600) (SCIEX, Concord, ON, Canada). Hydrophilic interaction liquid chromatography (HILIC) and reversed phase (RP) chromatography were used to analyze metabolites in positive and negative ion modes. A Waters ACQUITY UPLC@BEH Amide (100 × 2.1 mm, 1.7 µm) column was used in HILIC LC/MS at 30 °C with a flow rate of 0.3 mL/min. The injection volume was 5 μL. The mobile phase consisted of 5 mM ammonium formate and 0.02% formic acid in water (phase A) and 5 mM ammonium formate and 0.02% formic acid in acetonitrile: water (v:v=95:5) solution (phase B). The gradient was applied as follows: t = 0–2 min, 100% B; t = 9 min, 30% A and 70% B; t = 11 min, 70% A and 30% B; t = 13–18 min, 100% B. A Waters T3 (100 × 2.1 mm, 1.8 µm) column was employed in RP LC/MS at 40 °C with a flow rate of 0.4 mL/min and an injection volume of 5 μL. The mobile phase was composed of 2 mM ammonium formate and 0.02% formic acid in water (phase A) and the solution of acetonitrile: methanol (v:v=1:1) (phase B). The gradient was as follows: t = 0–0.5 min, 98% A and 2% B; t = 1.5 min, 80% A and 20% B; t = 4 min, 35% A and 65% B; t = 11–15 min, 5% A and 95% B; t = 15.1–18 min, 98% A and 2% B.

For mass spectrometric analysis, IDA-Top 10 collection mode was employed. The parameters were set as follows: CUR was 35; GS1 was 60; GS2 was 60; ISVF was 5,500 in positive mode and −4,500 in negative mode; TEM was 600; CE was 35; CES was 15; TOF MS mass range was 50–1,100 Da; TOF MS accumulation time was 0.120 s; TOF MS/MS mass range was 50–1,100 Da; TOF MS/MS accumulation time was 0.055 s.

MarkerView software (SCIEX) was used to perform principal component analysis-discriminant analysis (PCA-DA). We used the VIP (variable importance in the projection) value (threshold >1) and *p* value (threshold <0.05) of the *t*-test to evaluate the differential metabolites. The fold change of 1.5 was taken as the cutoff value. The database of *E. coli* K12 from KEGG was used to search metabolites.

### 2.5 Determination of specific growth rate

Cells were cultured in LB medium with 50 μg/mL carbenicillin at 37 °C, 200 rpm for 14 h. Cell suspension was then transferred to 1 mL M9 minimal medium, LB medium and TB medium, respectively, with the original OD_600 nm_ at 0.05. All media were supplemented with 50 μg/mL carbenicillin and 1 mM L-arabinose. Samples were cultured at 30 °C, 800 rpm in a microscreen high-throughput real-time microbial growth analysis system. OD_600 nm_ of the culture was detected every 30 min. Growth curve was fitted with Origin 9.1 according to the model of Schnute (Zwietering et al., 1990).

### 2.6 Flux balance analysis

The host strain used in this study was derived from BW25113 (*lacI* ^q^, *rrnB*_T14_, Δ*lacZ*_WJ16_, *hsdR*514, Δ*araBAD*_AH33_, Δ*rhaBAD*_LD78_) (Datsenko and Wanner, 2000) (Supplementary Table S1). We deleted the genes according to the genotype of BW25113 from model iML1515, the genome-scale reconstruction of metabolic network of *E. coli* K12 MG1655, by setting the upper bound and lower bound of the reactions to zero to construct the host strain (Monk et al., 2017) (Supplementary Table S3). In particular, there are three isoenzymes including gene *araD*-connected protein that catalyze the same reaction, so the reaction bounds of *ara*D remained unchanged. Gene *rhaA* is associated with two reactions, that is LYXI and RMI, and their lower and upper bounds were both set as zero. Strain C1 was constructed by knocking out genes *ldhA* and *dld*. On the basis of C1, strain C2 was constructed by adding the reaction to synthesize NCD (catalyzed by NcdS), and strain C3 was constructed by adding the reactions catalyzed by NcdS, *Ct*CTPS* and *Ft*NadE (Supplementary Table S4). Reactions were added according to the format of the model.

FBA was conducted with glucose uptake rate as the constraint under chemostat condition, and the maximum biomass reaction was set as the objective function. Glucose uptake rate was calculated according to Equation 1 as follows:

$$\text{glucose uptake rate}=\frac{c\times v}{M\times V\times OD600\times m}\text{mmol }g{DW}^{-1}h^{-1}$$ (1)

Where c is the concentration of glucose in the feeding medium (20 g/L), *v* is the medium feeding speed (15 mL/h), M is the molecular weight of glucose (180.16 g/mol), V is the working volume (300 mL), OD600 is the optical density of the culture at OD_600 nm_ (3.94, 3.92 and 3.83 for strain C1, C2 and C3, respectively), and m is cell dry weight per OD_600 nm_ (3 OD_600_ _nm_ = 1 gDW/L) (San et al., 2002).

Glucose uptake speeds were 4.23, 4.25 and 4.35 mmol gDW^-1^ h^-1^ for strain C1, C2 and C3, respectively. The Constraint-based reconstruction and analysis (COBRA) toolbox was used to construct engineered strains and perform FBA calculations (Heirendt et al., 2019). Reactions with flux higher than 10^–5^ and the fold change higher than 1.5 or lower than 0.67 were analyzed. Besides, we also paid attention to reactions with direction reversed. Fold change was calculated according to the reaction flux using strain C1 as the control.

### 2.7 ^13^C metabolic flux analysis

Strain C3 was cultured in LB medium with 50 μg/mL carbenicillin and 1 mM L-arabinose overnight at 37 °C, 200 rpm. Then cells were transferred to 50 mL of M9 medium containing 20 g/L glucose with the initial OD_600 nm_ of 0.01. The carbon source consisted of 80% unlabeled glucose and 20% [U-^13^C_6_] glucose (Cambridge Isotope Laboratories, Tewksbury, MA, United States). Cells were harvested when OD_600 nm_ reached 0.7–0.9 by centrifugation (14,000 *g*, 5 min, 4 °C). Both cell pellets and supernatants were collected for analysis. Strain BW25113 (Δ*ldhA, dld::cat*) was used as the control and cultured in the same way without the addition of carbenicillin and L-arabinose.

Glucose and organic acids in culture supernatants were analyzed using an ICS-5000^+^ series ion chromatography system (Thermo Fisher). For glucose quantification, the CarboPac PA100 column consisting of a guard column (4 × 50 mm) and an analytical column (4 × 250 mm) was employed. Samples were analyzed by an integrated pulsed amperometric detector. Isocratic elution with 20 mM NaOH and 10 mM sodium acetate as mobile phases was performed at 30 °C. The flow rate was set at 1.0 mL/min. For organic acid analysis, an IonPac AS11-HC column (Thermo Fisher) comprising a guard column (4 × 50 mm) and analytical column (4 × 250 mm) was used with a suppressed conductivity detector. Detections were carried out at 30 °C with 18 mM NaOH at the flow rate of 1.0 mL/min.

Cell pellets were hydrolyzed at 110 °C for 16 h in a heating block by adding 500 μL of 6 N HCl. The hydrolysates were cooled to room temperature and centrifuged at 14,000 *g* for 5 min to remove insoluble debris. Subsequently, 400 μL of the supernatant was transferred to 1.5 mL tubes, and was then dried under a gentle air stream at 65 °C using a nitrogen evaporator. For derivatization, 35 μL of anhydrous pyridine and 50 μL of N-(tert-butyldimethylsilyl)-N-methyl-trifluoroacetamide (MTBSTFA) containing 1% (w/w) tert-butyldimethylsilyl chloride (TBDMSCl) (Sigma-Aldrich) were added to the dried samples. The mixture was vortexed thoroughly and incubated at 60 °C for 30 min. Then the mixture was centrifuged at 14,000 *g* for 1 min, and 75 μL of the supernatant was used for GC-MS analysis (Long and Antoniewicz, 2019).

^13^C-metabolic flux analysis was performed using the Metran software platform (https://tlo.mit.edu/technologies/metran-software-13c-metabolic-flux-analysis), which implements the elementary metabolite unit (EMU) framework (Antoniewicz et al., 2007; Young et al., 2008). The central carbon metabolism model of *E. coli* K-12 MG1655 (BIGG iJO1366) (King et al., 2016) was used for flux fitting. Metabolic fluxes were estimated by minimizing the variance-weighted sum of squared residuals (SSR) between the experimentally measured and model predicted extracellular rates and mass isotopomer distributions of amino acids using nonlinear least-squares regression. To ensure the identification of global optimal solutions, the flux estimation procedure was systematically repeated using randomized initial flux values with a minimum of ten independent iterations. The statistical robustness of the estimated fluxes was verified through comprehensive sensitivity analysis, with 95% confidence intervals calculated by evaluating the response of the minimized SSR to systematic flux variations (Antoniewicz et al., 2006).

### 2.8 Cofactor extraction and determination

Strains were cultured in 2.5 mL TB medium at 30° C, 200 rpm for 23 h. A total of 1 × 10^10^ cells were collected and washed with 0.5 mL pre-cooled potassium phosphate (50 mM, pH 7.2), and then centrifuged at 10,000 *g*, 4 °C for 2 min. For the extraction of oxidized cofactors, 0.1 mL 0.2 M HCl was added, while 0.2 M NaOH was added for the extraction of reduced cofactors. Cells were resuspended and treated at 55 °C for 10 min, and then 0.1 mL 0.1 M NaOH or 0.1 mL 0.1 M HCl was added, respectively. Samples were centrifuged at 12,000 *g*, 4 °C for 15 min, and the supernatants were stored at −80 °C.

Cofactor concentrations were determined as follows: NCD(H) was detected in the volume of 0.1 mL with 50 mM HEPES (pH 7.5), 10 mM formate, 0.4 mM thiazolyl blue (MTT), 1 mM phenazine ethosulfate (PES), 0.27 mg/mL formate dehydrogenase mutant V198I/C256I/P260S/E261P/S381N/S383F (Guo et al., 2020a) and 10 μL of the cofactor sample. NAD(H) was detected in the volume of 0.1 mL with 100 mM Bicine (pH 8.0), 10 μL ethanol, 0.4 mM MTT, 1 mM PES, 4 mM EDTA, 5 U/mL yeast alcohol dehydrogenase II (Sigma-Aldrich) and 10 μL of the sample. NADP(H) was determined in the volume of 0.1 mL with 50 mM HEPES (pH 7.5), 10 mM fructose 1,6-bisphosphate (FBP), 0.4 mM MTT, 1 mM PES, 0.07 mg/mL fructose-bisphosphate aldolase class II from *E. coli* (FbaA) (Gene ID: 947,415), 0.05 mg/mL glyceraldehyde-3-phosphate dehydrogenase from *Streptococcus mutans* (*Sm*GAPN) (GenBank: AAN58410.1) and 10 μL of the sample. For the determination of the standard curves, the concentration of cofactors varied as follows (μM): 5, 10, 20, 40, 80, 160, 320. NCD was synthesized as reported (Wan et al., 2022). Reactions were carried out at 30 °C using a universal microplate spectrophotometer (BioTek, Winooski, VT, United States), and the absorbance at 570 nm was monitored continuously.

### 2.9 NCDH/NCD ratio regulation *in vivo*

Engineered strains were cultured in 7.5 mL LB with 50 μg/mL carbenicillin and kanamycin at 37 °C, 200 rpm for 10 h. Then 0.5 mL of the seed culture was transferred to 50 mL TB medium with 50 μg/mL carbenicillin, 50 μg/mL kanamycin, 0.1 mM IPTG and 1 mM L-arabinose. Cells were cultured at 30 °C, 200 rpm for 19 h. A total of 1 × 10^10^ cells were collected by centrifugation and washed with 0.3 mL 40 mM MOPS (pH 6.5). Subsequently, cell pellets were resuspended in 0.5 mL M9 medium with 40 mM MOPS, 50 μg/mL carbenicillin, 50 μg/mL kanamycin, 0.1 mM IPTG and 1 mM L-arabinose, and were cultured at 30° rC, 200 rpm for 20 min. Formate with concentration at 15 mM was supplied in the medium as needed. Finally, cell pellets were collected and used for cofactor extraction. Cofactor concentration was determined as described above. All samples were prepared in triplicate.

### 2.10 Protein purification

All proteins were expressed in BL21 (DE3) and purified with the Ni-NTA kit (Invitrogen, Carlsbad, CA, United States). Specifically, strains were cultured in LB medium with 50 μg/mL kanamycin (for *Sm*GAPN) or 34 μg/mL chloramphenicol (for FbaA) at 37 °C for 12 h. Then the culture was transferred to TB medium with 0.1 mM IPTG and corresponding antibiotics, and cultured at 30 °C for 48 h. For *Sm*GAPN, 3 mM β-mercaptoethanol was added in buffers in the purification steps. For FbaA, 0.3 mM ZnCl_2_ was supplemented in TB medium. FDH mutants were purified as reported (Guo et al., 2020b). Protein concentration was measured with a Bio-Rad protein assay kit using BSA as standard. SDS-PAGE was conducted to analyze protein purity.

## 3 Results

### 3.1 Strain construction and cofactor level determination

To determine the effects of NCD biosynthesis, we first constructed a group of *E. coli* strains that can synthesize NCD at varying concentrations (Supplementary Table S1). Briefly, we constructed plasmids that expressed the NCD synthetase NcdS alone and NcdS with additional enzymes *Ft*NadE and *Ct*CTPS* (Wang et al., 2021). These plasmids were transformed into the host strain to give the strains C2 and C3, respectively. For comparison, the control strain C1 was obtained upon transformation with the parental empty plasmid. These strains were cultured in terrific broth (TB) medium, and cells were collected for cofactor extraction. Then intracellular cofactor concentrations of the engineered strains were determined. In strain C3, NCD concentration reached 2.77 mM, while only 0.14 mM NCD was produced in strain C2. As expected, no NCD was detected in the control strain C1 (Figure 1C). Moreover, as cellular NCD level increased, NAD(P) level decreased, indicating a competitive relationship in the synthesis of these cofactors. Therefore, these strains, C1 devoid of NCD production, C2 and C3, capable of producing NCD at micromolar level and millimolar level, respectively, offered a perfect set to evaluate the global effects of the newly-introduced process of NCD biosynthesis.

### 3.2 Comparative proteomics analysis

We first documented the proteomic responses to NCD biosynthesis. Samples from chemostat culture of *E. coli* cells were collected for proteomics analysis. Total proteins were extracted, digested and labeled with tandem mass tags (TMT) method successively (Thompson et al., 2003), and then were detected by capillary high performance liquid chromatography-mass spectrometry. *Escherichia coli* K12 MG1655 proteome (UniProt ID UP000000625) was used as the reference database with the addition of overexpressed proteins in engineered strains (Supplementary Table S2). A total of 25,444 peptides were identified and were further mapped to 3 overexpressed proteins (*Ct*CTPS*, *Ft*NadE and NcdS) and 2,371 endogenous proteins (Supporting Data 1), accounting for 53.3% of the database and the vast majority of the expressed proteome (Mori et al., 2021; Schmidt et al., 2016). Compared with strain C1, there were 82 proteins upregulated and 98 downregulated in strain C3, and 55 proteins upregulated and 12 downregulated in strain C2. These differentially expressed proteins constituted 7.6% and 2.8% of the identified proteins in strain C3 and C2, respectively, and accounted for 4.0% and 1.5% of the total proteome. These data displayed a positive correlation with NCD concentration.

Gene Ontology (GO) analysis revealed that in the C3/C1 group (comparing strain C3 to strain C1), proteins associated with binding, catalytic activity, protein-containing complex, transporter activity, cellular anatomical entity, response to stimulus, localization and biological regulation were disturbed with both up- and downregulation (Figure 2A). For the C2/C1 group, similar processes were perturbed, but more proteins participating in transporter activity and localization were upregulated, while the downregulated proteins were not significantly enriched. These results indicated that similar processes were affected in strains with different NCD levels, and that enhanced NCD biosynthesis included slightly more protein changes. This prompted us to analyze the shared differentially expressed proteins in group C3/C1 and C2/C1. There were 27 proteins identified in both groups (Figure 2B). Among them, the up- and downregulated proteins with the highest fold change were nonessential, such as AraC, FecA, FecB and Gmm (Baba et al., 2006; Keseler et al., 2021) (Supporting Data 2), which are associated with membrane-related transport and sugar metabolism.

**FIGURE 2:**
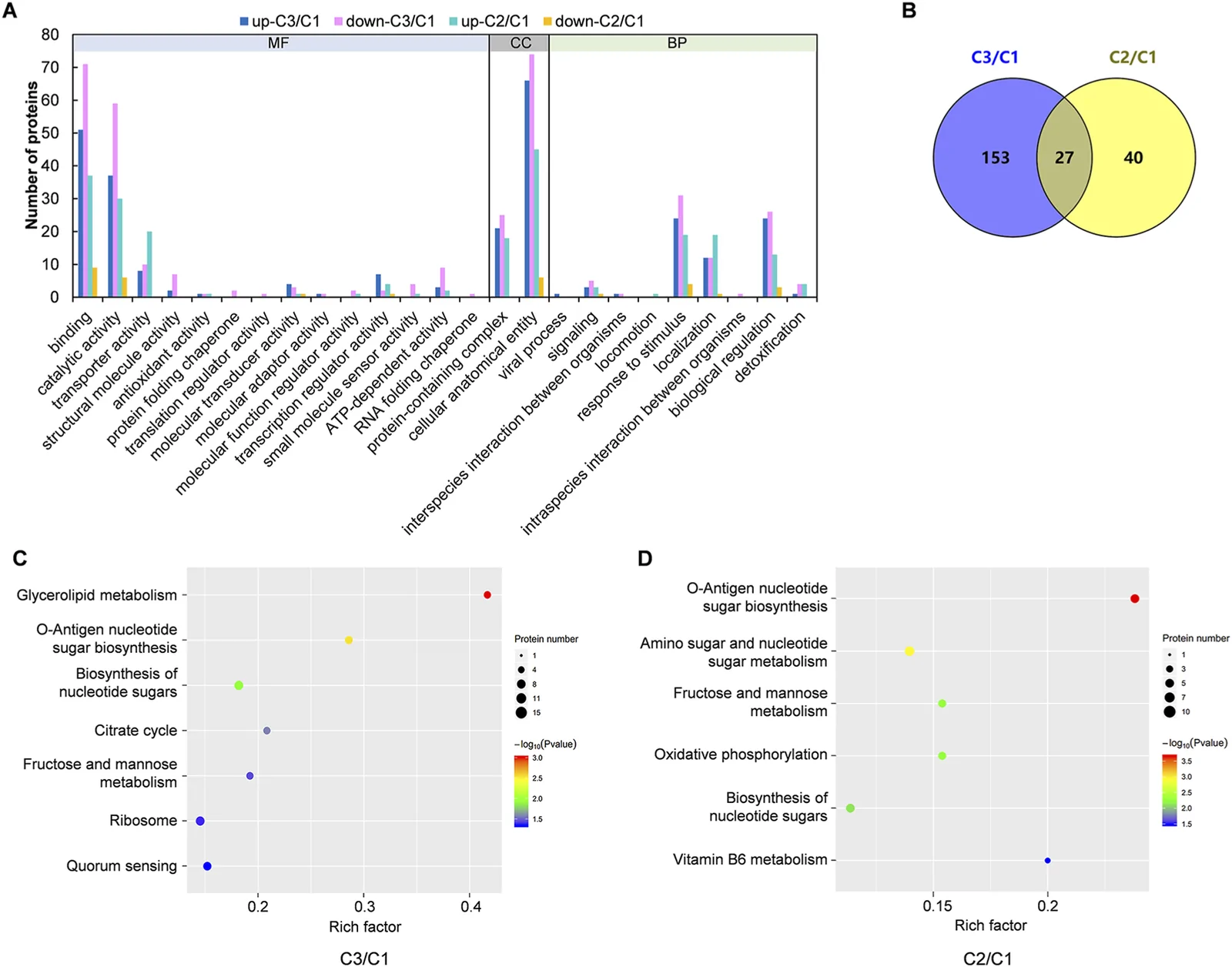
Proteomic analyses of NCD self-sufficient strains (C3 and C2) and the control strain (C1). **(A)** Gene ontology annotation of up- and downregulated proteins. MF, molecular function; CC, cellular component; BP, biological process. **(B)** Venn diagram analysis of differentially expressed proteins between group C3/C1 and C2/C1. **(C)** and **(D)**, KEGG enrichment of differentially expressed proteins in group C3/C1 and C2/C1.

As to the overall profile of differentially expressed proteins, we performed KEGG enrichment analysis. O-antigen nucleotide sugar biosynthesis, biosynthesis of nucleotide sugars, and fructose and mannose metabolism were enriched in both groups (Figures 2C,D). Additionally, glycerolipid metabolism in group C3/C1 showed the highest proportion of differentially expressed proteins, reaching 41.7% (5 of 12) (Supporting Data 3). Other enriched pathways contained fewer differentially expressed proteins with proportions below 30%. It was notable that, in group C3/C1, 5 of 24 proteins were differentially expressed in the TCA cycle (Figure 2C and Supporting Data 3). Specifically, Icd, AcnA, Mqo and FrdA were upregulated and FumC was downregulated. Nevertheless, all these proteins displayed less than 2-fold changes (Supporting Data 1). It should be noted that those proteins are involved in biochemical reactions catalyzed by isozymes (Supplementary Figure S2). These data implied that limited pathways were significantly enriched in NCD biosynthesis strains. Hence, we speculated that proteins being differentially expressed had their corresponding functions compensated by reactions catalyzed by isozymes. Thus, it seemed that the NCD biosynthesis associated disturbance of protein abundance was largely mitigated through cellular self-regulation.

### 3.3 Global metabolomics analysis

To get more insights into cellular responses to NCD biosynthesis, we next conducted metabolomics analysis. Both hydrophilic interaction liquid chromatography and reversed phase chromatography were employed to analyze metabolites in positive and negative ion modes. Principal components analysis was performed to assess the metabolic differences. For group C3/C1, a total of 170 metabolites were identified, and 65 of them exhibited significant changes. Specifically, 24 metabolites increased and 41 decreased. For the group C2/C1, 147 metabolites were identified, and fewer differential metabolites were observed compared to group C3/C1. There were 14 increased metabolites and 8 decreased metabolites (Supporting Data 4).

We further analyzed significantly differential metabolites in group C3/C1 and C2/C1. In strains that synthesized NCD, metabolites involved in (*R*)-pantothenate metabolism and glycerophospholipid metabolism exhibited higher concentration than those of the control strain C1, while the amounts of *L*-homocysteine and *O*-succinyl-*L*-homoserine in *L*-methionine synthesis decreased (Figure 3A). Compared to strain C1, *sn*-glycero-3-phosphocholine in glycerophospholipid pathway showed a sharp increase in strain C2 and C3 with fold changes approaching 20. Besides, in group C3/C1, metabolites in *O*-antigen nucleotide sugar biosynthesis and sugar metabolism decreased slightly.

**FIGURE 3:**
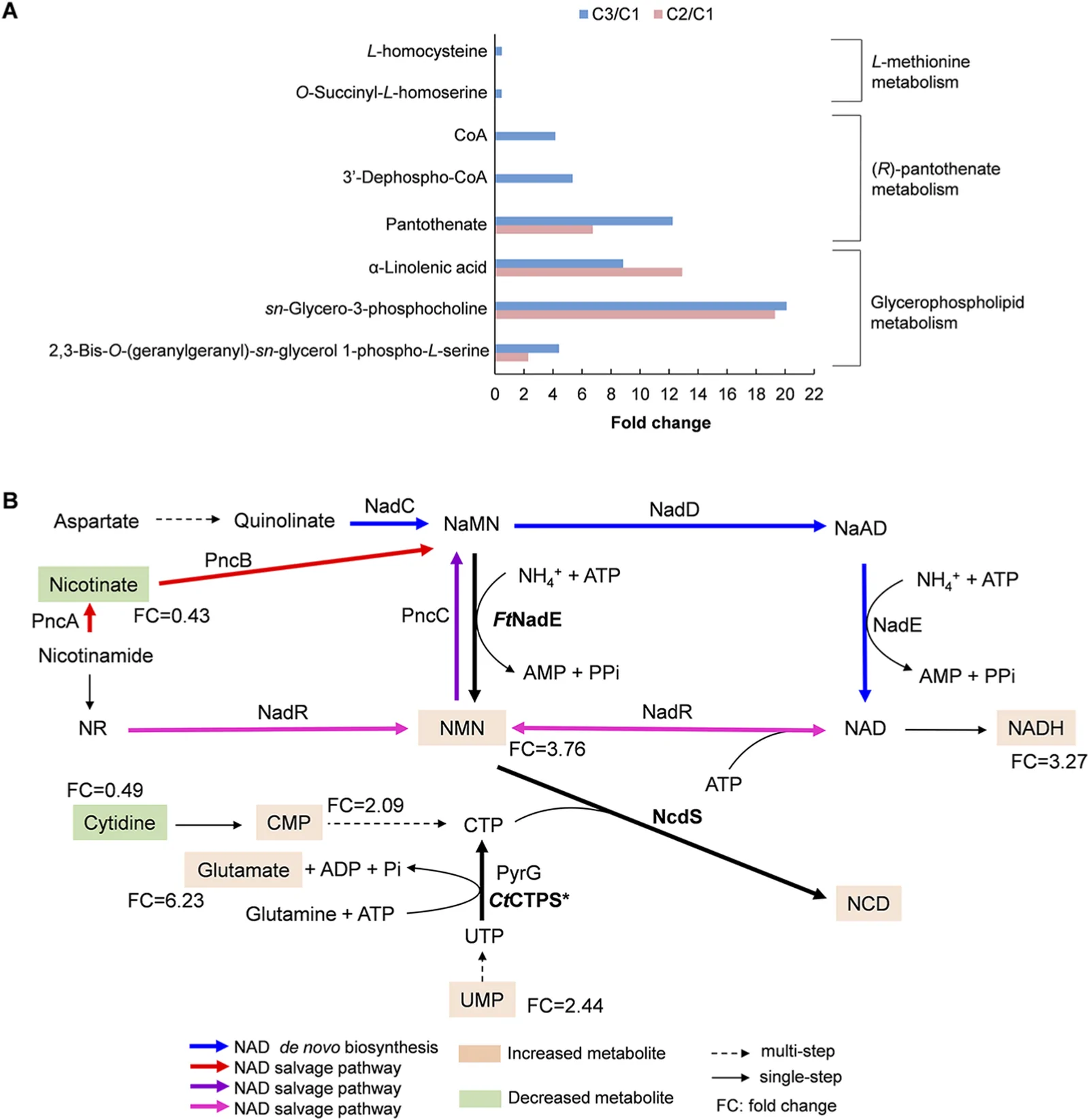
Metabolic changes of group C3/C1 and C2/C1. **(A)** Differential metabolites in L-methionine metabolism (*R*)-pantothenate and glycerophospholipid metabolism in sample group C3/C1 and C2/C1. **(B)** Nucleotide metabolism changes in group C3/C1. C3, strain with millimolar level of NCD; C2, strain with micromolar level of NCD; C1, strain devoid of NCD biosynthesis. *Ct*CTPS*, CTP synthetase mutant *Ct*CTPS* (D149 E) derived from *C*. *trachomatis*; *Ft*NadE, NMN synthetase from *F*. *tularensis*; NadC, quinolinate phosphoribosyltransferase; NadD, nicotinate-nucleotide adenylyltransferase; NadE, NH_3_-dependent NAD synthetase; NadR, NMN adenylyltransferase; NcdS, NCD synthetase; PncA, nicotinamidase; PncB, nicotinate phosphoribosyltransferase; PncC, NMN amidohydrolase; PyrG, CTP synthetase; AMP, adenosine monophosphate; ATP, adenosine triphosphate; CMP, cytidine monophosphate; CTP, cytidine triphosphate; NaAD, nicotinate adenine dinucleotide; NAD, nicotinamide adenine dinucleotide; NADH, reduced NAD; NaMN, *β*-nicotinate *D*-ribonucleotide; NCD, nicotinamide cytosine dinucleotide; NMN, nicotinamide mononucleotide; NR, Nicotinamide riboside; Pi, phosphate; PPi, pyrophosphate; UMP, uridine monophosphate.

As NCD is synthesized from nicotinamide mononucleotide (NMN) and CTP, and both of them are nucleotide metabolites (Figure 1A), we next focused on related metabolites therein. In the group C3/C1 and C2/C1, NCD levels increased as expected, and the concentrations of its precursors CMP and UMP were enhanced. Glutamate exhibited a 6.23-fold increase in C3 compared to C1 (Figure 3B), while no obvious change was observed in the group C2/C1 (Supplementary Figure S3 and Supporting Data 4). It can be noted that overexpression of *Ct*CTPS* in strain C3 promoted the synthesis of CTP with the concurrent production of glutamate. The reaction that synthesizes NCD needs more NMN and CTP which is consistent with the downregulation of NMN in strain C2 without this precursor enhanced. Compared with strain C1, nicotinate level decreased in strain C3 but remained unchanged in C2. In strain C2 with micromolar level of NCD, the concentrations of both *β*-nicotinate *D*-ribonucleotide (NaMN) and NMN dropped due to metabolic flux shunt to NCD. Also, nicotinamide riboside (NR) level declined on account of its phosphorylation to NMN. In strain C3 with millimolar level of NCD, more NMN was synthesized from NaMN which enhanced the conversion from nicotinate to NaMN. The above results indicate that in NCD self-sufficient strains, besides the *de novo* biosynthetic pathway from aspartate, the salvage pathways leading to NaMN and NMN also play key roles in supplying NMN for NCD synthesis, which is in fact competing with NAD synthesis (Gazzaniga et al., 2009; Kurnasov et al., 2002).

### 3.4 *In silico* flux balance analysis

To further unravel the effect of NCD biosynthesis, we performed *in silico* flux balance analysis (FBA) of the engineered strains (Orth et al., 2010). We used iML1515, the genome-scale reconstruction of metabolic network in *E. coli* K12 MG1655, as the metabolic model for flux balance calculation (Monk et al., 2017). The model iML1515 was first modified by deleting the genes according to the genotype of the host strain and adding the reactions linked to NCD synthesis to give strains C1, C2 and C3 (Supplementary Tables S3 and S4).

FBA was conducted using the Constraint-Based Reconstruction and Analysis (COBRA) toolbox (Heirendt et al., 2019). The theoretical growth rates of strain C1, C2 and C3 were determined to be 0.37 h^-1^, 0.36 h^-1^ and 0.37 h^-1^, respectively. Compared to strain C1, there were 8 reactions absent and 6 reactions emerged in strain C2. However, no reactions were determined as differential. In strain C3, 32 reactions were removed and 32 new reactions appeared in comparison with strain C1. It is noteworthy that reactions catalyzed by NcdS and *Ft*NadE were not shown in strain C3 despite the overexpression of these two proteins. We speculated that this was because these two reactions had little contribution to biomass production. Reactions with absolute flux higher than 0.01 were further analyzed (Figure 4; Supplementary Figure S4). Downregulated reactions were mainly involved in purine and pyrimidine metabolism, alternate carbon metabolism and the transmembrane transportation of fumarate. Upregulated reactions were associated with purine and pyrimidine metabolism, alternate carbon metabolism, nucleotide salvage pathway, histidine metabolism and organic acid transport. In strain C1, endogenous CTP synthetase (PyrG) exhibited a flux of 0.057 for CTP synthesis. In strain C3, overexpression of *Ct*CTPS* led to a flux of 0.059 with the endogenous flux reduced to zero (Figure 4A). This indicated that in strain C3, CTP was exclusively derived from the catalysis of *Ct*CTPS*, which is free from product inhibition (Endrizzi et al., 2004; Wylie et al., 1996).

**FIGURE 4:**
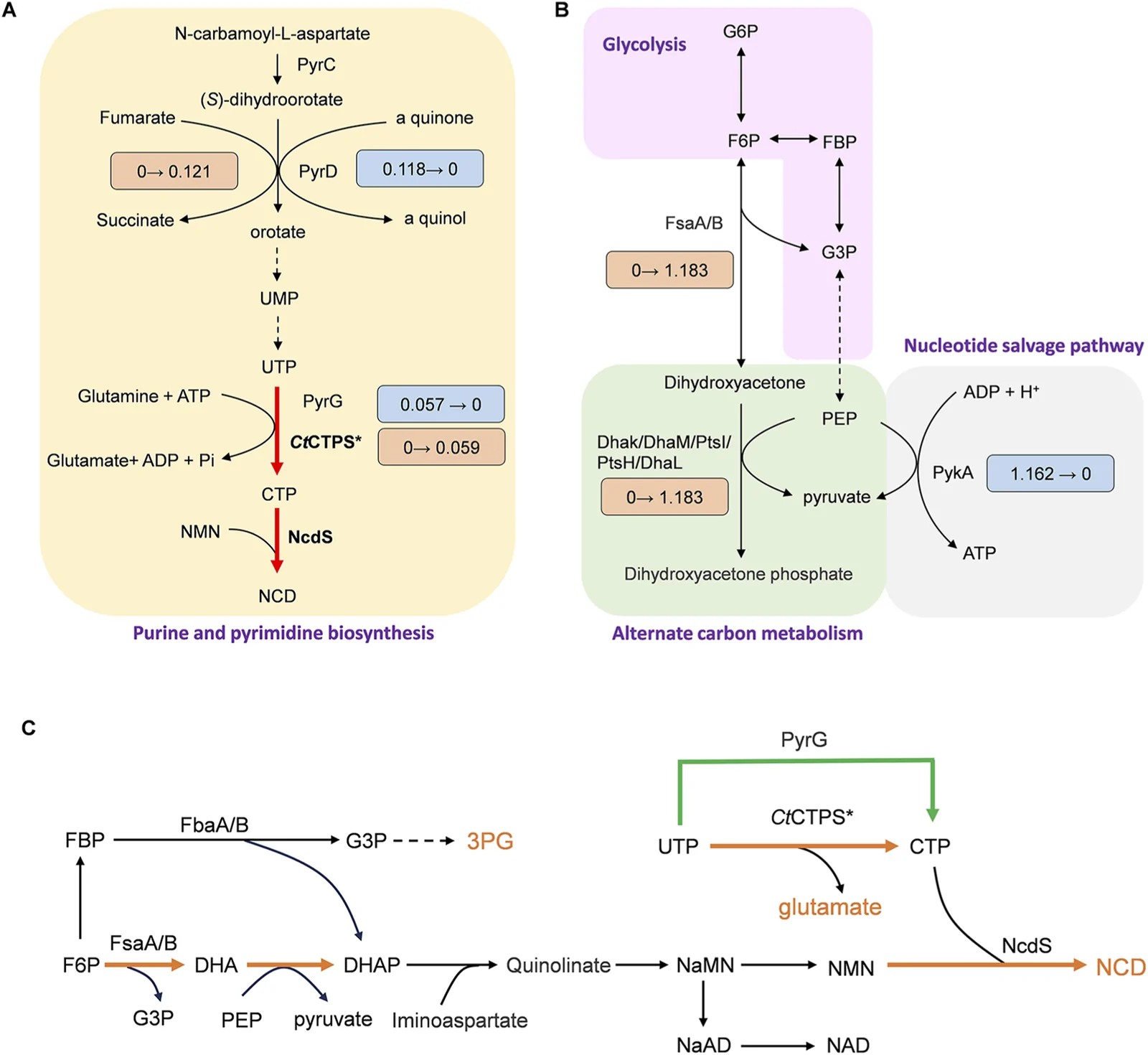
Flux changes of strain C3 compared to strain C1 according to FBA. **(A)** Purine and pyrimidine biosynthesis. **(B)** Glycolysis, alternate carbon metabolism and nucleotide salvage pathway. Adk, adenylate kinase; *Ct*CTPS, CTP synthetase mutant *Ct*CTPS* (D149 E) derived from *C*. *trachomatis*; DhaK, dihydroxyacetone kinase subunit K; DhaL, dihydroxyacetone kinase subunit L; DhaM, dihydroxyacetone kinase, phosphotransferase component; FbaA/B, fructose-bisphosphate aldolase I and II; FsaA/B, fructose-6-phosphate aldolase I and II; PtsH, phosphocarrier protein HPr; PtsI, PTS enzyme I; PykA, pyruvate kinase 2; PyrC, dihydroorotase; PyrD, Dihydroorotate dehydrogenase; PyrG, CTP synthetase; CTP, cytidine triphosphate; DHAP, dihydroxyacetone phosphate; FBP, fructofuranose 1,6-bisphosphate; F6P, fructose 6-phosphate; G3P, glyceraldehyde 3-phosphate; G6P, D-glucose 6-phosphate; NAD, nicotinamide adenine dinucleotide; NaAD, nicotinate adenine dinucleotide; NaMN, β-nicotinate D-ribonucleotide; NCD, nicotinamide cytosine dinucleotide; NMN, nicotinamide mononucleotide; PEP, phosphoenolpyruvate; UTP, uridine triphosphate; 3 PG, 3-phospho-D-glycerate. Flux changes are shown in blue and orange boxes. **(C)** Analysis of flux balance data and omics data. Orange arrows represent reactions with flux upregulated; green arrows represent reactions with flux downregulated. Orange words represent increased metabolites.

Significantly, the reaction catalyzed by F6P aldolase (FsaA/B) was activated in strain C3, which generated more flux to dihydroxyacetone and D-glyceraldehyde 3-phosphate (G3P) (Figure 4B). Concurrently, the flux to the formation of dihydroxyacetone phosphate (DHAP) was upregulated, enhancing the biosynthesis of NCD through the *de novo* biosynthetic pathway shared with NAD (Zhu et al., 2021). In *E. coli*, the transformation of F6P is mainly accomplished through glycolysis with fructose-bisphosphate aldolase (FbaA/B), since the cleavage of F6P is more endergonic than the fructose-bisphosphate cleavage reaction (Figure 4C) (Riemer et al., 2013). However, the phosphorylation of dihydroxyacetone is expected to circumvent this problem and promote the cleavage of F6P (Schürmann and Sprenger, 2001). The flux for DHAP synthesis increased, whereas the DHAP pool did not change significantly, suggesting that the generated DHAP was rapidly consumed. Given that DHAP is a precursor for NCD synthesis, it is speculated that the activation of FsaA/B may serve to replenish DHAP consumed in the NCD synthetic pathway. We also found that a network was formed according to the omics data and flux balance analysis. The increased flux driven by *Ct*CTPS* was in agreement with the increased concentration of glutamate (Figures 3B, 4C).

### 3.5 Mapping the global responses to NCD biosynthesis reveals four metabolic nodes

We next characterized strain C2 and C3 by combining the omics and flux analysis data to clarify cellular responses to NCD biosynthesis. It was found that sugar metabolism and methionine synthesis were downregulated. Meanwhile, pyrimidine-related synthesis, pantothenate and CoA synthesis, phospholipid metabolism, isocitrate, arginine and phenylalanine were upregulated (Figure 5A). Specifically, the increased flux from F6P to DHAP, which was supposed to be used for NCD synthesis, led to the downregulation of sugar-related metabolism (Supplementary Figure S5). Both protein abundance and the metabolite levels were shown to be reduced. For example, dTDP-4-acetamido-4,6-dideoxy-α-D-galactose in *O*-antigen nucleotide sugar biosynthesis decreased by 12.5-fold, and most proteins in the same pathway were downregulated. Hence, F6P, connected to central carbon metabolism, is the first branch node in NCD self-sufficient strains.

**FIGURE 5:**
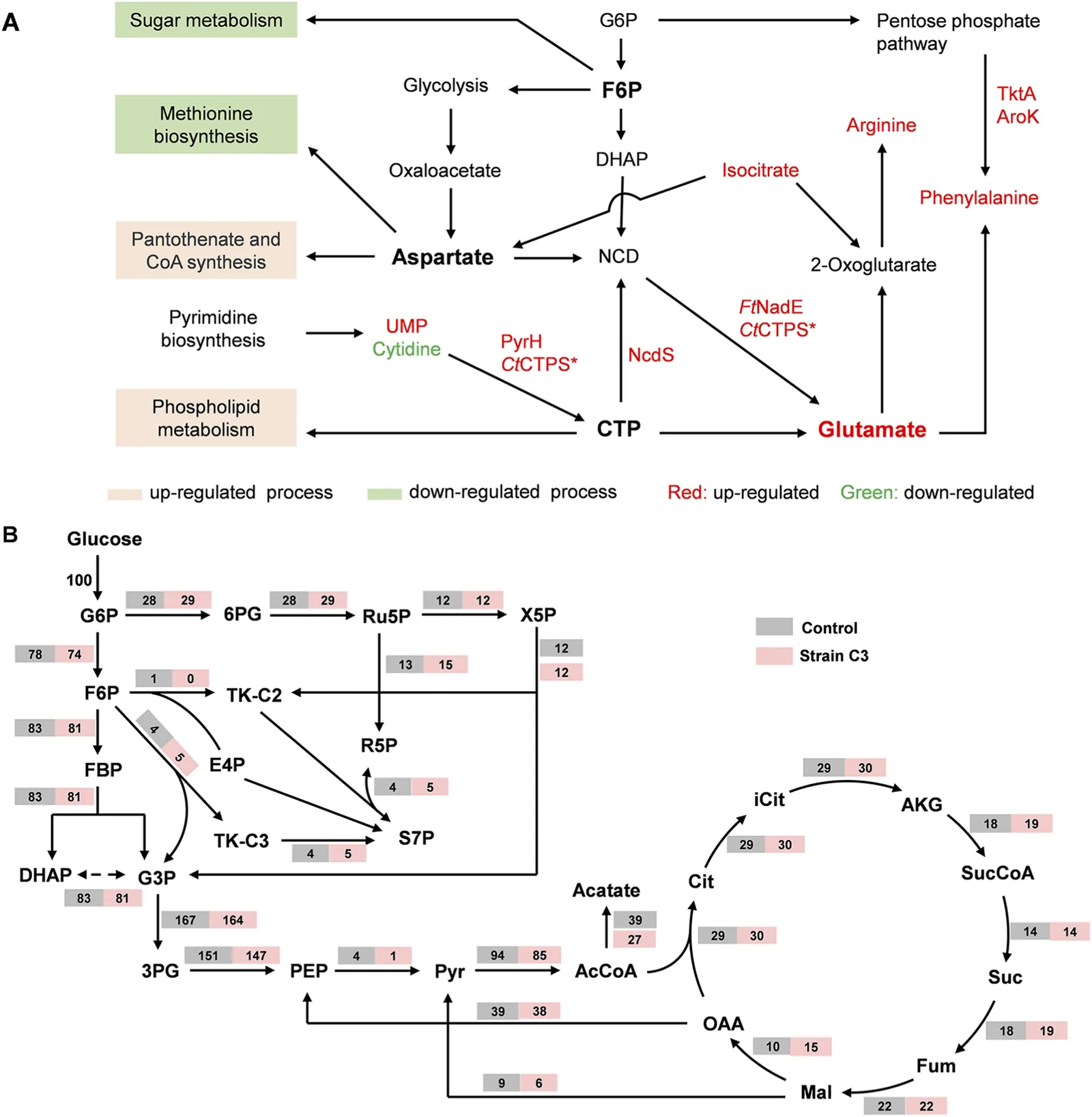
The overall cellular responses to NCD biosynthesis **(A)** and metabolic flux analysis **(B)**. AroK, shikimate kinase 1; *Ct*CTPS*, CTP synthetase mutant (D149 E) derived from *C*. *trachomatis*; *Ft*NadE, nicotinamide mononucleotide synthetase from *F*. *tularensis*; NcdS, NCD synthetase; PyrH, UMP kinase; TktA, transketolase 1. For metabolic flux analysis, cells were cultured in 50 mL of M9 medium containing 20 g/L glucose (80% natural glucose and 20% [U-^13^C_6_] glucose) at 37 °C, 200 rpm with initial OD_600 nm_ at 0.01. Samples were collected when the OD_600 nm_ of the culture reached 0.7–0.9, and then were prepared for GC-MS analysis. E4P, erythrose 4-phosphate; Ru5P, ribulose 5-phosphate; R5P, ribose 5-phosphate; S7P, sedoheptulose 7-phosphate; TK-C2, transketolase for C2 unit; TK-C3, transketolase for C3 unit; X5P, xylulose 5-phosphate; 6 PG, 6-phosphate gluconate.

On the other hand, CTP was identified as the second branch node. The synthesis of phospholipids like *sn*-glycero-3-phosphocholine was enhanced dramatically. We speculated that this was attributed to the increased flux to DHAP and the enhanced synthesis of CTP (Supplementary Figure S6). UMP kinase (PyrH) was upregulated and promoted the conversion of UMP to UDP (Reaves et al., 2013). This process further facilitated the synthesis of UTP which was then transformed to CTP by *Ct*CTPS*. Besides, the disturbance of pyrimidine biosynthesis affected the metabolism of sugar and phospholipid, since the diphosphate derivatives of purine and pyrimidine participate in the synthesis of sugars, glycoproteins and phospholipids (Parsons and Rock, 2013). Given the special role of CTP in phospholipid metabolism (Zhu et al., 2025), NCD is connected to phospholipid-related pathways and rewires the metabolic network in a small degree.

According to the metabolic network, aspartate is involved in the *de novo* synthesis of NCD, the synthesis of methionine and CoA, and also connected to the TCA cycle. The downregulation of methionine biosynthesis and the upregulation of pantothenate and CoA synthesis show that aspartate is the third branch node resulting from its extra allocation in NCD synthesis. The shikimate pathway, which is the upstream of aromatic amino acids (Zhao and Winkler, 1994), is linked to glycolysis through the pentose phosphate pathway (PPP). The upregulation of transketolase 1 (TktA) and shikimate kinase 1 (AroK) promotes the synthesis of shikimate and chorismate, and thus results in the upregulation of phenylalanine. Besides, overexpression of *Ct*CTPS* increases the amount of glutamate which benefits the synthesis of phenylalanine (Figure 5A). This demonstrates that glutamate is the fourth key branch node of the disturbance.

To evaluate the central metabolism, we next conducted metabolic flux analysis of strain C3 based on ^13^C-labeling technology (Figure 5B). Strain C1 harbors the empty plasmid but does not synthesize NCD, and is metabolically equivalent to BW25113 (Δ*ldhA*, *dld::cat*). BW25113 (Δ*ldhA*, *dld::cat*) was used as the control to provide a valid baseline for comparing flux changes in central metabolism in strain C3. Compared to the control strain, glycolysis and PPP of strain C3 exhibited similar carbon fluxes, indicating that NCD synthesis caused no significant perturbation on these two pathways. Notably, the fluxes from pyruvate to acetyl-CoA and from acetyl-CoA to acetate decreased by approximately 10% in strain C3. It is speculated that more flux was oriented to oxaloacetate and then aspartate to synthesize NCD (Figure 5A). These moderate perturbations on central metabolism prompted us to evaluate the growth profiles of NCD self-sufficient strains.

More NCD self-sufficient strains were constructed using different hosts *E. coli* DH5α and DH10B (Supplementary Table S1). Expression of NcdS in DH5α and DH10B produced strains XJC02 and XJC06, respectively. Control strains XJC01 and XJC05 were generated by transforming the empty plasmid into their corresponding hosts. Thus, similar to the strain group C1, C2 and C3 with BW25113 as the host, the DH5α group consisted of strains XJC01, XJC02 (expressing NcdS) and XJC03 (expressing NcdS plus *Ft*NadE and *Ct*CTPS*), and the DH10B group included strains XJC05, XJC06 (expressing NcdS) and XJC07 (expressing NcdS plus *Ft*NadE and *Ct*CTPS*) (Wang et al., 2021). Cells were cultured in M9 minimal medium, LB medium and TB medium, respectively, and OD_600 nm_ of the culture was monitored. It showed that in the same medium, strain C1, C2 and C3 exhibited comparable specific growth rates and similar lag phases (Supplementary Table S5). Cells grew fastest in nutrient-rich TB medium and slowest in M9 medium. Similar phenomena were observed with strains in the DH5α and DH10B groups. Notably, these specific growth rates were consistent with the growth rates calculated by FBA; that is, strains C1, C2 and C3 exhibited similar growth rates. Therefore, each group of cells showed similar growth profiles in different media regardless of the incorporation of different NCD biosynthetic modules. These results suggested that NCD biosynthesis has no detrimental effects on cell growth and that NCD is biologically compatible.

### 3.6 Rational tuning of cellular NCDH/NCD ratio

Finally, we tried to tune cellular NCDH/NCD ratio and evaluate potential crosstalk with those of natural redox cofactors. In *E. coli*, NADH/NAD and NADPH/NADP redox couples are involved in energy metabolism and the maintenance of redox balance (Xiao et al., 2018). Under aerobic growth conditions, the concentration of NADH is lower than that of NAD with an NADH/NAD ratio of as low as 0.03 (Park et al., 2016). Under certain conditions, this ratio varies and can approach about 1 (Liu et al., 2019b; Yang et al., 2024). In contrast, intracellular concentration of NADPH is usually higher than that of NADP, and the NADPH/NADP ratio maintains at around 1.5 (Park et al., 2016; Yang et al., 2024). Fluctuation in either NADH/NAD ratio or NADPH/NADP ratio disrupts redox homeostasis and further causes complex biological effects (Vemuri et al., 2006). In NCD self-sufficient *E. coli* strains, NCDH/NCD acts as a non-natural redox couple that participates in the selected reactions (Guo et al., 2020b; Wang et al., 2021).

We tuned the NCDH/NCD ratio by using an NCD-preferring formate dehydrogenase (FDH*) to verify the independent redox tunability of NCD (Guo et al., 2020b). Engineered strain XJD2201-1 was constructed by overexpressing FDH* and NCD biosynthetic module (containing NcdS, *Ft*NadE and *Ct*CTPS*) (Figure 6A). It was found that NCDH concentration increased remarkably and NCDH/NCD ratio reached 5.2 upon treatment with formate (Figure 6B). This indicated that 84% of total NCD(H) existed as the reduced form. On the other hand, the ratios of NADH/NAD and NADPH/NADP were much lower. NADPH/NADP ratio remained unchanged when adding formate in the culture, while the ratio of NADH/NAD increased slightly. We speculated that this was likely due to the residual activity of FDH* to NAD (Guo et al., 2020b). On the other hand, this observation was in alignment with the fact that cellular redox homeostasis is tightly controlled (Vemuri et al., 2006; Yang et al., 2024). These results demonstrated the redox states of natural cofactors were basically isolated from the fluctuation of NCDH/NCD ratio. Moreover, these data further confirm that NCD is a biocompatible molecule and may be used as a separated redox cofactor for specific reactions.

**FIGURE 6:**
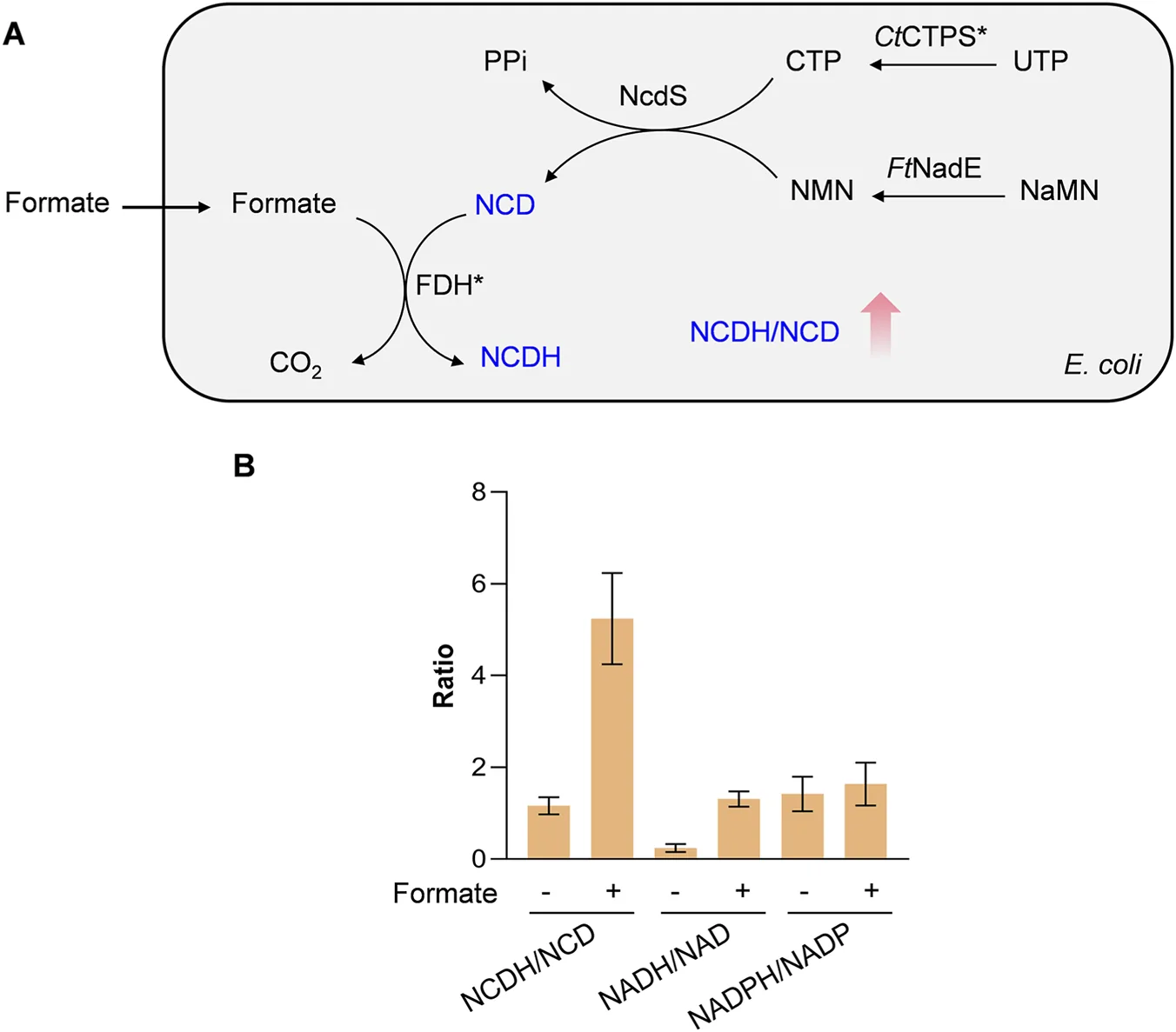
Tuning intracellular NCDH/NCD ratio in engineered strains. **(A)** Scheme to regulate NCDH/NCD ratio. FDH*, formate dehydrogenase mutant V198I/C256I/P260S/E261P/S381N/S383F. **(B)** Ratios of NCDH/NCD, NADH/NAD and NADPH/NADP. The data are presented as mean ± SD (n = 3). Source data are provided as a Source Data file. Strains were cultured in LB medium supplemented with 50 μg/mL carbenicillin and kanamycin at 37 °C, 200 rpm for 10 h, and then aliquots of the culture were transferred to TB medium supplemented with the same antibiotics, 0.1 mM IPTG and 1 mM L-arabinose. Cells were cultured at 30 °C, 200 rpm for 19 h, and then were transferred to M9 medium containing 40 mM MOPS (pH 6.5), 50 μg/mL carbenicillin and kanamycin, 0.1 mM IPTG, 1 mM L-arabinose and 15 mM formate, followed by incubation at 30 °C, 200 rpm for 20 min.

## 4 Discussion

In this study, we elucidated cellular responses to NCD biosynthesis by employing systematic analysis of NCD self-sufficient cells capable of producing NCD up to millimolar levels. Proteomics analysis showed that similar proteins were affected regardless of NCD at different levels, and nonessential proteins and those involved in reactions with isozymes were disturbed. Metabolomics analysis revealed minor changes in sugar metabolism, methionine synthesis (*R*)-pantothenate metabolism and glycerophospholipid metabolism. Importantly, it was indicated that both the *de novo* NAD biosynthesis pathway and the salvage pathways contribute NMN for NCD synthesis. Moreover, FBA analysis unveiled that NCD synthesis induced only a few flux redistributions and affected subordinate reactions. By combining these observations, the global responses to NCD biosynthesis were mapped, and four metabolic nodes, namely, F6P, CTP, aspartate and glutamate, were discovered, which depicted the connections of disturbed processes. Finally, we demonstrated the successful upregulation of the NCDH/NCD ratio by the expression of an NCD-preferring formate dehydrogenase, while the ratios between reduced and oxidized natural redox cofactors showed little fluctuation. Most importantly, we constructed several lines of *E. coli* cells that could synthesize NCD at different levels and confirmed that these cells grew similarly to their parental strains in either synthetic minimal media or nutrient-rich media. Overall, these results suggested that NCD biosynthesis is well-compatible with endogenous metabolism and that the NCDH/NCD ratio can be tuned independently with little consequence in terms of the redox states of natural nicotinamide cofactors.

It should be noted that the current study could not distinguish the biological effects of NCD *per se* and NCD biosynthesis. While it is valuable to acquire cellular responses to NCD without the introduction of its biosynthetic enzymes, this is technically challenging as cells are known incapable of taking up nicotinamide cofactors from media (Reidl et al., 2002). Previously, we constructed *E. coli* strains to assimilate NAD and NCD by overexpressing different nucleotide transporters and found that the engineered strains suffered from abnormal growth (Wang et al., 2019; Zhou et al., 2011), indicating that the transporter itself gave complicated consequences.

Nonetheless, we can now consider broader applications of NCD in synthetic biology and beyond. Over the years, redox cofactor engineering to supply reducing power has been regarded as a pivotal strategy to improve the production of target compounds in engineered strains (Wang et al., 2013; Wu et al., 2019; Ye et al., 2023). Unlike natural cofactors whose regulation can cause global redox imbalances, NCD may serve as an alternative cofactor that mediates the biosynthesis of targeted compounds (Bat-Erdene et al., 2021; Lan et al., 2023; Zhang et al., 2025; Zhu et al., 2022). By expressing NCD synthetase in different hosts, more NCD self-sufficient strains are expected to be constructed as microbial cell factories. It is worth noting that NAD(H) and NADP(H) participate in numerous biochemical reactions, and concentration fluctuations of these nicotinamide cofactors may trigger feedback inhibition. Furthermore, excessive NADH can induce cytotoxicity by promoting the production of reactive oxygen species. As an artificially designed molecule, NCD exhibits fewer or negligible interactions with endogenous enzymes compared with the natural cofactors. Thus, modulating the NCDH/NCD ratio allows pathway-specific control with reduced risk of triggering native feedback inhibition or natural cofactor toxicity, conferring enhanced robustness to the regulatory system.

As a non-natural redox cofactor, NCD shows promise for use in energy regeneration and cellular energy regulation. With NCD-preferring enzymes, NCD can be employed to concentrate intracellular energy and reconstruct energy metabolism, thereby promoting the generation of ATP or other energy-rich compounds. It is hopeful that ATP might be regenerated *in vivo* by using NCD *in lieu* of NAD(P) as the cofactor (Willett and Banta, 2023). Recently, a malic enzyme-based system was constructed to transfer electrons from natural cofactors to NCD, and lactate was synthesized with NCDH (Xue et al., 2026), demonstrating that energy was converged to the specific reaction through NCDH. Further, the utilization of NCD may lead to the development of new energy supply modes to develop advanced cell factories for C1 biotransformations (Cowan et al., 2025; Orsi et al., 2024). Moreover, given that NCD shares structural and functional similarities with NAD, we speculate that it may be applied as a new tool to study aging-related processes including DNA repair and autophagy (Covarrubias et al., 2020; Li et al., 2017).

Taken together, the results at systems level clarified the biological effects of NCD biosynthesis and confirmed the segregation feature of NCD in terms of redox regulation *in vivo*, which will facilitate a broader application of NCD as a non-natural cofactor in chemical biology and synthetic biology. In the future, NCD may be used to regulate cellular energy metabolism and to create more efficient and robust biotechnological systems.

## Data Availability

The mass spectrometry proteomics data have been deposited to the ProteomeXchange Consortium (https://proteomecentral.proteomexchange.org) via the iProX partner repository (Chen T et al., 2022) with the dataset identifier PXD064813. The metabolomics raw data are available at the NIH Common Fund’s National Metabolomics Data Repository (NMDR) website, the Metabolomics Workbench (https://www.metabolomicsworkbench.org) with the Project ID PR002930.

## References

[B1] An J. Chen T. Ge F. Zhang W. Li L. Tang B. (2025). Dual-channel energy pathway combining energy molecule supply and electron transfer to support solar-to-chemical production in an *E. Coli*–thylakoid hybrid. Nat. Synth. 4, 1408–1421. 10.1038/s44160-025-00853-0

[B2] Antoniewicz M. R. Kelleher J. K. Stephanopoulos G. (2006). Determination of confidence intervals of metabolic fluxes estimated from stable isotope measurements. Metab. Eng. 8 (4), 324–337. 10.1016/j.ymben.2006.01.004 16631402

[B3] Antoniewicz M. R. Kelleher J. K. Stephanopoulos G. (2007). Elementary metabolite units (EMU): a novel framework for modeling isotopic distributions. Metab. Eng. 9 (1), 68–86. 10.1016/j.ymben.2006.09.001 17088092 PMC1994654

[B4] Aspacio D. Zhang Y. Cui Y. Luu E. King E. Black W. B. (2024). Shifting redox reaction equilibria on demand using an orthogonal redox cofactor. Nat. Chem. Biol. 20 (11), 1535–1546. 10.1038/s41589-024-01702-5 PMC12316058 39138383

[B5] Baba T. Ara T. Hasegawa M. Takai Y. Okumura Y. Baba M. (2006). Construction of *Escherichia coli* K-12 in-frame, single-gene knockout mutants: the keio collection. Mol. Syst. Biol. 2, 2006.0008. 10.1038/msb4100050 16738554 PMC1681482

[B6] Bat-Erdene U. Billingsley J. M. Turner W. C. Lichman B. R. Ippoliti F. M. Garg N. K. (2021). Cell-free total biosynthesis of plant terpene natural products using an orthogonal cofactor regeneration system. ACS Catal. 11 (15), 9898–9903. 10.1021/acscatal.1c02267 PMC8963176 35355836

[B7] Black W. B. Perea S. Li H. (2023). Design, construction, and application of noncanonical redox cofactor infrastructures. Curr. Opin. Biotechnol. 84, 103019. 10.1016/j.copbio.2023.103019 37939631

[B8] Chen R. Gao J. Yu W. Chen X. Zhai X. Chen Y. (2022). Engineering cofactor supply and recycling to drive phenolic acid biosynthesis in yeast. Nat. Chem. Biol. 18 (5), 520–529. 10.1038/s41589-022-01014-6 35484257

[B9] Chen T. Ma J. Liu Y. Chen Z. Xiao N. Lu Y. (2022). iProX in 2021: connecting proteomics data sharing with big data. Nucleic Acids Res. 50 (D1), D1522–D1527. 10.1093/nar/gkab1081 PMC8728291 34871441

[B10] Covarrubias A. J. Perrone R. Grozio A. Verdin E. (2020). NAD^+^ metabolism and its roles in cellular processes during ageing. Nat. Rev. Mol. Cell Biol. 22 (2), 119–141. 10.1038/s41580-020-00313-x PMC7963035 33353981

[B11] Cowan A. E. Hillers M. Rainaldi V. Collas F. Choudhary H. Zakaria B. S. (2025). Fast growth and high-titer bioproduction from renewable formate via metal-dependent formate dehydrogenase in *Escherichia coli* . Nat. Commun. 16 (1), 5908. 10.1038/s41467-025-61001-y PMC12218308 40593698

[B12] Datsenko K. A. Wanner B. L. (2000). One-step inactivation of chromosomal genes in *Escherichia coli* K-12 using PCR products. Proc. Natl. Acad. Sci. U. S. A. 97 (12), 6640–6645. 10.1073/pnas.120163297 10829079 PMC18686

[B13] Endrizzi J. A. Kim H. Anderson P. M. Baldwin E. P. (2004). Crystal structure of *Escherichia coli* cytidine triphosphate synthetase, a nucleotide-regulated glutamine amidotransferase/ATP-dependent amidoligase fusion protein and homologue of anticancer and antiparasitic drug targets. Biochemistry 43, 6447–6463. 10.1021/bi0496945 15157079 PMC2891762

[B14] Gazzaniga F. Stebbins R. Chang S. Z. McPeek M. A. Brenner C. (2009). Microbial NAD metabolism: lessons from comparative genomics. Microbiol. Mol. Biol. Rev. 73 (3), 529–541. 10.1128/mmbr.00042-08 19721089 PMC2738131

[B15] Ge J. Wang T. Yu H. Ye L. (2025). *De novo* biosynthesis of nylon 12 monomer ω-aminododecanoic acid. Nat. Commun. 16 (1), 175. 10.1038/s41467-024-55739-0 PMC11695860 39747160

[B16] Guo X. Liu Y. Wang Q. Wang X. Li Q. Liu W. (2020a). Non-natural cofactor and formate-driven reductive carboxylation of pyruvate. Angew. Chem. Int. Ed. 59 (8), 3143–3146. 10.1002/anie.201915303 31845497

[B17] Guo X. Wang X. Liu Y. Li Q. Wang J. Liu W. (2020b). Structure-guided design of formate dehydrogenase for regeneration of a non-natural redox cofactor. Chem. Eur. J. 26 (70), 16611–16615. 10.1002/chem.202003102 32815230

[B18] Heirendt L. Arreckx S. Pfau T. Mendoza S. N. Richelle A. Heinken A. (2019). Creation and analysis of biochemical constraint-based models using the COBRA toolbox v.3.0. Nat. Protoc. 14 (3), 639–702. 10.1038/s41596-018-0098-2 30787451 PMC6635304

[B19] Ji D. Wang L. Hou S. Liu W. Wang J. Wang Q. (2011). Creation of bioorthogonal redox systems depending on nicotinamide flucytosine dinucleotide. J. Am. Chem. Soc. 133 (51), 20857–20862. 10.1021/ja2074032 22098020

[B20] Katsyuba E. Romani M. Hofer D. Auwerx J. (2020). NAD^+^ homeostasis in health and disease. Nat. Metab. 2 (1), 9–31. 10.1038/s42255-019-0161-5 32694684

[B21] Keseler I. M. Gama-Castro S. Mackie A. Billington R. Bonavides-Martínez C. Caspi R. (2021). The EcoCyc database in 2021. Front. Microbiol. 12, 711077. 10.3389/fmicb.2021.711077 PMC8357350 34394059

[B22] King Z. A. Lu J. Drager A. Miller P. Federowicz S. Lerman J. A. (2016). BiGG models: a platform for integrating, standardizing and sharing genome-scale models. Nucleic Acids Res. 44 (D1), D515–D522. 10.1093/nar/gkv1049 26476456 PMC4702785

[B23] Kissman E. N. Sosa M. B. Millar D. C. Koleski E. J. Thevasundaram K. Chang M. C. Y. (2024). Expanding chemistry through *in vitro* and *in vivo* biocatalysis. Nature 631 (8019), 37–48. 10.1038/s41586-024-07506-w 38961155

[B24] Kurnasov O. V. Polanuyer B. M. Ananta S. Sloutsky R. Tam A. Gerdes S. Y. (2002). Ribosylnicotinamide kinase domain of nadr protein: identification and implications in NAD biosynthesis. J. Bacteriol. 184 (24), 6906–6917. 10.1128/jb.184.24.6906-6917.2002 12446641 PMC135457

[B25] Lan H.-N. Liu R.-Y. Liu Z.-H. Li X. Li B.-Z. Yuan Y.-J. (2023). Biological valorization of lignin to flavonoids. Biotechnol. Adv. 64, 108107. 10.1016/j.biotechadv.2023.108107 36758651

[B26] Lee S. Y. Kim H. U. Chae T. U. Cho J. S. Kim J. W. Shin J. H. (2019). A comprehensive metabolic map for production of bio-based chemicals. Nat. Catal. 2 (1), 18–33. 10.1038/s41929-018-0212-4

[B27] Li J. Bonkowski M. S. Moniot S. Zhang D. Hubbard B. P. Ling A. J. Y. (2017). A conserved NAD^+^ binding pocket that regulates protein-protein interactions during aging. Science 355, 1312–1317. 10.1126/science.aad8242 28336669 PMC5456119

[B28] Lim S. Banta S. (2025). Engineering noncanonical cofactors to expand cellular functions. ACS Synth. Biol. 14 (9), 3321–3331. 10.1021/acssynbio.5c00473 40923492

[B29] Liu Y. Feng Y. Wang L. Guo X. Liu W. Li Q. (2019a). Structural insights into phosphite dehydrogenase variants favoring a non-natural redox cofactor. ACS Catal. 9, 1883–1887. 10.1021/acscatal.8b04822

[B30] Liu Y. Landick R. Raman S. (2019b). A regulatory NADH/NAD^+^ redox biosensor for bacteria. ACS Synth. Biol. 8 (2), 264–273. 10.1021/acssynbio.8b00485 30633862

[B31] Liu Y. Cong Y. Zhang C. Fang B. Pan Y. Li Q. (2021a). Engineering the biomimetic cofactors of NMNH for cytochrome P450 BM3 based on binding conformation refinement. RSC Adv. 11 (20), 12036–12042. 10.1039/d1ra00352f PMC8696588 35423749

[B32] Liu Y. Luo C. Li T. Zhang W. Zong Z. Liu X. (2021b). Reduced nicotinamide mononucleotide (NMNH) potently enhances NAD^+^ and suppresses glycolysis, the TCA cycle, and cell growth. J. Proteome Res. 20 (5), 2596–2606. 10.1021/acs.jproteome.0c01037 33793246

[B33] Long C. P. Antoniewicz M. R. (2019). High-resolution ^13^C metabolic flux analysis. Nat. Protoc. 14 (10), 2856–2877. 10.1038/s41596-019-0204-0 31471597

[B34] Meng D. Liu M. Su H. Song H. Chen L. Li Q. (2023). Coenzyme engineering of glucose-6-phosphate dehydrogenase on a nicotinamide-based biomimic and its application as a glucose biosensor. ACS Catal. 13 (3), 1983–1998. 10.1021/acscatal.2c04707

[B35] Monk J. M. Lloyd C. J. Brunk E. Mih N. Sastry A. King Z. (2017). iML1515, a knowledgebase that computes *Escherichia coli* traits. Nat. Biotechnol. 35 (10), 904–908. 10.1038/nbt.3956 29020004 PMC6521705

[B36] Montaño López J. Duran L. Avalos J. L. (2021). Physiological limitations and opportunities in microbial metabolic engineering. Nat. Rev. Microbiol. 20 (1), 35–48. 10.1038/s41579-021-00600-0 34341566

[B37] Mori M. Zhang Z. Banaei‐Esfahani A. Lalanne J. B. Okano H. Collins B. C. (2021). From coarse to fine: the absolute *Escherichia coli* proteome under diverse growth conditions. Mol. Syst. Biol. 17 (5), e9536. 10.15252/msb.20209536 PMC8144880 34032011

[B38] Orsi E. Hernández-Sancho J. M. Remeijer M. S. Kruis A. J. Volke D. C. Claassens N. J. (2024). Harnessing noncanonical redox cofactors to advance synthetic assimilation of one-carbon feedstocks. Curr. Opin. Biotechnol. 90, 103195. 10.1016/j.copbio.2024.103195 39288659

[B39] Orth J. D. Thiele I. Palsson B. O. (2010). What is flux balance analysis? Nat. Biotechnol. 28 (3), 245–248. 10.1038/nbt.1614 20212490 PMC3108565

[B40] Park J. O. Rubin S. A. Xu Y. F. Amador-Noguez D. Fan J. Shlomi T. (2016). Metabolite concentrations, fluxes and free energies imply efficient enzyme usage. Nat. Chem. Biol. 12 (7), 482–489. 10.1038/nchembio.2077 27159581 PMC4912430

[B41] Parsons J. B. Rock C. O. (2013). Bacterial lipids: metabolism and membrane homeostasis. Prog. Lipid Res. 52 (3), 249–276. 10.1016/j.plipres.2013.02.002 23500459 PMC3665635

[B42] Paul C. E. Arends I. W. C. E. Hollmann F. (2014). Is simpler better? Synthetic nicotinamide cofactor analogues for redox chemistry. ACS Catal. 4 (3), 788–797. 10.1021/cs4011056

[B43] Reardon S. (2024). How synthetic biologists are building better biofactories. Nature 628, 224–226. 10.1038/d41586-024-00907-x 38561408

[B44] Reaves M. L. Young B. D. Hosios A. M. Xu Y.-F. Rabinowitz J. D. (2013). Pyrimidine homeostasis is accomplished by directed overflow metabolism. Nature 500 (7461), 237–241. 10.1038/nature12445 23903661 PMC4470420

[B45] Reidl J. Schlör S. Kraiß A. Schmidt‐Brauns J. Kemmer G. Soleva E. (2002). NADP and NAD utilization in *Haemophilus influenzae* . Mol. Microbiol. 35 (6), 1573–1581. 10.1046/j.1365-2958.2000.01829.x 10760156

[B46] Richardson K. N. Black W. B. Li H. (2020). Aldehyde production in crude lysate- and whole cell-based biotransformation using a noncanonical redox cofactor system. ACS Catal. 10 (15), 8898–8903. 10.1021/acscatal.0c03070 PMC8294662 34306803

[B47] Riemer S. A. Rex R. Schomburg D. (2013). A metabolite-centric view on flux distributions in genome-scale metabolic models. BMC Syst. Biol. 7, 33. 10.1186/1752-0509-7-33 23587327 PMC3644240

[B48] San K. Y. Bennett G. N. Berrios-Rivera S. J. Vadali R. V. Yang Y. T. Horton E. (2002). Metabolic engineering through cofactor manipulation and its effects on metabolic flux redistribution in *Escherichia coli* . Metab. Eng. 4 (2), 182–192. 10.1006/mben.2001.0220 12009797

[B49] Schmidt A. Kochanowski K. Vedelaar S. Ahrne E. Volkmer B. Callipo L. (2016). The quantitative and condition-dependent *Escherichia coli* proteome. Nat. Biotechnol. 34 (1), 104–110. 10.1038/nbt.3418 26641532 PMC4888949

[B50] Schürmann M. Sprenger G. A. (2001). Fructose-6-phosphate aldolase is a novel class I aldolase from *Escherichia coli* and is related to a novel group of bacterial transaldolases. J. Biol. Chem. 276, 11055–11061. 10.1074/jbc.M008061200 11120740

[B51] Thompson A. Schafer J. Kuhn K. Kienle S. Schwarz J. Schmidt G. (2003). Tandem mass tags: a novel quantification strategy for comparative analysis of complex protein mixtures by MS/MS. Anal. Chem. 75, 1895–1904. 10.1021/ac0262560 12713048

[B52] Tian Z. Liu L. Wu L. Yang Z. Zhang Y. Du L. (2024). Enhancement of vitamin B6 production driven by omics analysis combined with fermentation optimization. Microb. Cell Fact. 23 (1), 137. 10.1186/s12934-024-02405-1 PMC11095007 38750497

[B53] Tran V. G. Tan S.-I. Xu H. Weilandt D. R. Li X. Bhagwat S. S. (2025). Decompartmentalization of the yeast mitochondrial metabolism to improve chemical production in *Issatchenkia orientalis* . Nat. Commun. 16 (1), 7110. 10.1038/s41467-025-62304-w PMC12318132 40753087

[B54] Vemuri G. N. Altman E. Sangurdekar D. P. Khodursky A. B. Eiteman M. A. (2006). Overflow metabolism in *Escherichia coli* during steady-state growth: transcriptional regulation and effect of the redox ratio. Appl. Environ. Microbiol. 72 (5), 3653–3661. 10.1128/AEM.72.5.3653-3661.2006 16672514 PMC1472329

[B55] Wan L. Wang X. Hu Y. Li Q. Zhao Z. K. (2022). Gram-scale biocatalytic preparation of the non-natural cofactor nicotinamide cytosine dinucleotide. Tetrahedron Lett. 88, 153568. 10.1016/j.tetlet.2021.153568

[B56] Wang Y. San K. Y. Bennett G. N. (2013). Cofactor engineering for advancing chemical biotechnology. Curr. Opin. Biotechnol. 24 (6), 994–999. 10.1016/j.copbio.2013.03.022 23611567

[B57] Wang L. Ji D. Liu Y. Wang Q. Wang X. Zhou Y. J. (2017). Synthetic cofactor-linked metabolic circuits for selective energy transfer. ACS Catal. 7 (3), 1977–1983. 10.1021/acscatal.6b03579

[B58] Wang L. Liu B. Liu Y. Sun Y. Liu W. Yu D. (2019). *Escherichia coli* strain designed for characterizing *in vivo* functions of nicotinamide adenine dinucleotide analogues. Org. Lett. 21 (9), 3218–3222. 10.1021/acs.orglett.9b00935 30995052

[B59] Wang X. Feng Y. Guo X. Wang Q. Ning S. Li Q. (2021). Creating enzymes and self-sufficient cells for biosynthesis of the non-natural cofactor nicotinamide cytosine dinucleotide. Nat. Commun. 12 (1), 2116. 10.1038/s41467-021-22357-z PMC8035330 33837188

[B60] Wang P. Zhou H. Y. Zhou J. P. Li B. Liu Z. Q. Zheng Y. G. (2022). Module engineering coupled with omics strategies for enhancing D-pantothenate production in *Escherichia coli* . Bioresour. Technol. 352, 127024. 10.1016/j.biortech.2022.127024 35337996

[B61] Willett E. Banta S. (2023). Synthetic NAD(P)(H) cycle for ATP regeneration. ACS Synth. Biol. 12 (7), 2118–2126. 10.1021/acssynbio.3c00172 37369039

[B62] Wu W. Zhang Y. Liu D. Chen Z. (2019). Efficient mining of natural NADH-utilizing dehydrogenases enables systematic cofactor engineering of lysine synthesis pathway of *Corynebacterium glutamicum* . Metab. Eng. 52, 77–86. 10.1016/j.ymben.2018.11.006 30458240

[B63] Wylie J. L. Wang L. L. Tipples G. McClarty G. (1996). A single point mutation in CTP synthetase of *Chlamydia trachomatis* confers resistance to cyclopentenyl cytosine. J. Biol. Chem. 271 (26), 15393–15400. 10.1074/jbc.271.26.15393 8663065

[B64] Xiao W. Wang R. S. Handy D. E. Loscalzo J. (2018). NAD(H) and NADP(H) redox couples and cellular energy metabolism. Antioxid. Redox Signal. 28 (3), 251–272. 10.1089/ars.2017.7216 28648096 PMC5737637

[B65] Xu J.-Z. Yang H.-K. Zhang W.-G. (2018). NADPH metabolism: a survey of its theoretical characteristics and manipulation strategies in amino acid biosynthesis. Crit. Rev. Biotechnol. 38 (7), 1061–1076. 10.1080/07388551.2018.1437387 29480038

[B66] Xue H. Hu Y. Shah A. M. Wang X. Guo X. Huang Y. (2026). Malic enzyme-based system for transhydrogenation between nicotinamide cofactors. Synth. Syst. Biotechnol. 11, 10–18. 10.1016/j.synbio.2025.08.009 PMC12492669 41050200

[B67] Yang J. Wu Y. Lv X. Liu L. Li J. Du G. (2024). Engineering redox cofactor balance for improved 5-methyltetrahydrofolate production in *Escherichia coli* . J. Agric. Food Chem. 72 (17), 9974–9983. 10.1021/acs.jafc.4c00821 38625685

[B68] Ye M. Gao J. Zhou Y. J. (2023). Global metabolic rewiring of the nonconventional yeast *Ogataea polymorpha* for biosynthesis of the sesquiterpenoid β-elemene. Metab. Eng. 76, 225–231. 10.1016/j.ymben.2023.02.008 36828231

[B69] Young J. D. Walther J. L. Antoniewicz M. R. Yoo H. Stephanopoulos G. (2008). An elementary metabolite unit (EMU) based method of isotopically nonstationary flux analysis. Biotechnol. Bioeng. 99 (3), 686–699. 10.1002/bit.21632 17787013

[B70] Zhang W. Zhang H. Shang Y. Luo Y. Wu H. Wu H. (2025). High-yield biosynthesis of 3-hydroxypropionic acid from acetate in metabolically engineered *Escherichia coli* . ACS Synth. Biol. 14 (5), 1654–1666. 10.1021/acssynbio.5c00030 40267313

[B71] Zhao G. Winkler M. E. (1994). An *Escherichia coli* K-12 tktA tktB mutant deficient in transketolase activity requires pyridoxine (vitamin B6) as well as the aromatic amino acids and vitamins for growth. J. Bacteriol. 176 (19), 6134–6138. 10.1128/jb.176.19.6134-6138.1994 7928977 PMC196835

[B72] Zhou Y. Wang L. Yang F. Lin X. Zhang S. Zhao Z. K. (2011). Determining the extremes of the cellular NAD(H) level by using an *Escherichia coli* NAD(+)-auxotrophic mutant. Appl. Environ. Microbiol. 77 (17), 6133–6140. 10.1128/AEM.00630-11 21742902 PMC3165392

[B73] Zhu F. Peña M. Bennett G. N. (2021). Metabolic engineering of *Escherichia coli* for quinolinic acid production by assembling L-aspartate oxidase and quinolinate synthase as an enzyme complex. Metab. Eng. 67, 164–172. 10.1016/j.ymben.2021.06.007 PMC10024596 34192552

[B74] Zhu F. Liu D. Chen Z. (2022). Recent advances in biological production of 1,3-propanediol: new routes and engineering strategies. Green Chem. 24 (4), 1390–1403. 10.1039/d1gc04288b

[B75] Zhu Y. Tong X. Xue J. Qiu H. Zhang D. Zheng D.-Q. (2025). Phospholipid biosynthesis modulates nucleotide metabolism and reductive capacity. Nat. Chem. Biol. 21 (1), 35–46. 10.1038/s41589-024-01689-z 39060393

[B76] Zwietering M. H. Jongenburger I. Rombouts F. M. Riet K. V. t. (1990). Modeling of the bacterial growth curve. Appl. Environ. Microbiol. 56 (6), 1875–1881. 10.1128/aem.56.6.1875-1881.1990 16348228 PMC184525

