# Supplementary material for: Multi-omics analyses reveal cellular responses to biosynthesis of the non-natural cofactor nicotinamide cytosine dinucleotide
Source: Front Bioeng Biotechnol. 2026 Sep 29;14:1954596. doi: 10.3389/fbioe.2026.1954596 (PMC13658176; doi:10.3389/fbioe.2026.1954596)
Supplement: Supplementary file 1 [file Supplementaryfile2.docx]

Supplementary Material

**Multi-omics analyses reveal cellular responses to biosynthesis of the non-natural cofactor nicotinamide cytosine dinucleotide**

Xiaojia Guo^1^*, Qian Wang^2^, Ting Liu^3^, Yinghan Hu^2^, Xueying Wang^2^, Zongbao K. Zhao^1^*

^1^MOE Key Laboratory of Bio-Intelligent Manufacturing, School of Bioengineering, Dalian University of Technology, Dalian, China.

^2^Laboratory of Biotechnology, Dalian Institute of Chemical Physics, CAS, Dalian, China.

^3^SCIEX. China, Shanghai, China.

Number of supplementary figures: 6.

Number of supplementary tables: 5.

**1. Supplementary Figures and Tables**

## 1.1 Supplementary Figures

**Supplementary Figure S1.** Residual glucose concentration (A) and OD600 nm (B) of engineered strains during chemostat cultivation.

**Supplementary Figure S2.** TCA cycle in E. coli. *acn*AB, aconitate hydratase AB; *fum*ABCDE, fumarase ABCDE; *frd*ABCD, fumarate reductase ABCDE; *icd*, isocitrate dehydrogenase; *lpd*, lipoamide dehydrogenase; *mae*A, NAD-dependent malic enzyme; *mdh*, malate dehydrogenase; *mqo*, malate:quinone oxidoreductase; *suc*ABCD, succinyl-CoA synthetase.

**Supplementary Figure S3.** **Nucleotide metabolism changes in group C2/C1.** NadC, quinolinate phosphoribosyltransferase; NadD, nicotinate-nucleotide adenylyltransferase; NadE, NH_3_-dependent NAD synthetase; NadR, NMN adenylyltransferase; NcdS, NCD synthetase; PncA, nicotinamidase; PncB, nicotinate phosphoribosyltransferase; PncC, NMN amidohydrolase; PyrG, CTP synthetase; AMP, adenosine monophosphate; ATP, adenosine triphosphate; CMP, cytidine monophosphate; CTP, cytidine triphosphate; NaAD, nicotinate adenine dinucleotide; NAD, nicotinamide adenine dinucleotide; NADH, reduced NAD; NaMN, β-nicotinate D-ribonucleotide; NCD, nicotinamide cytosine dinucleotide; NMN, nicotinamide mononucleotide; NR, nicotinamide riboside; Pi, phosphate; PPi, pyrophosphate; UMP, uridine monophosphate.

**Supplementary Figure S4. Flux changes of strain C3 compared to strain C1 in histidine metabolism, alternate carbon metabolism (A) and transport in inner membrane (B).** DauA, aerobic C4-dicarboxylate transporter; DcuA, C4-dicarboxylate transporter; DcuB, anaerobic C4-dicarboxylate transporter; DcuC, anaerobic C4-dicarboxylate transporter; DeoB, phosphopentomutase; PhnN, ribose 1,5-bisphosphate phosphokinase; Prs, ribose-phosphate diphosphokinase.

**Supplementary Figure S5. Down-regulated processes related to D-fructose 6-phosphate.** Dashed arrows represent multiple steps. FC, fold change.

**Supplementary Figure S6. Up-regulated processes associated with dihydroxyacetone and CTP in NCD self-sufficient strains.** Dashed arrows represent multiple steps. FC, fold change.

## 1.2 Supplementary Tables

**Supplementary Table S1.** Strains and plasmids used in this study.

| Strains and plasmids | Characteristic | Source |
| --- | --- | --- |
| **Strains** |  |  |
| BW25113 | *lacIq, rrnBT*14*, ΔlacZWJ*16*, hsdR5*14*,* Δ*araBADAH*33*,* Δ*rhaBADLD*78 | (Datsenko and Wanner 2000) |
| BL21(DE3) | F^-^, *dcm, ompT, hsdS (*rB^-^*,* mB^-^*), gal, λ*(DE3) | Novagen |
| DH5α | F-, φ*80d/lacZ*ΔM15, Δ*(lacZYA-argF)*U169, *deoR*, *recA*1, *endA*1, *hsdR*17*(*rk-, mk+*)*, *phoA*, *supE*44, *λ-*, *thi-1*, *gyrA*96, *relA*1 | TaKaRa |
| DH10B | F-, *endA*1, *deoR*, *recA*1, *galE*15, *galK*16, *nupG, rpsL*Δ*(lac)X*74, φ80*lacZ*ΔM15, *araD139,* Δ*(ara, leu)*7697, *mcrA* Δ*(mrr-hsdRMS-mcrBC*), Str^R^, λ | Invitrogen |
| BW25113 (*△ldhA, dld::cat*) | BW25113*,* Δ*ldhA, dld::cat* | (Wang et al. 2021) |
| C1 | BW25113 (Δ*ldhA, dld::cat*), pUC-P15A | This study |
| C2 | BW25113 (Δ*ldhA, dld::cat*), pUC-P15A-NCDS | This study |
| C3 (XYC5017) | BW25113 (Δ*ldhA, dld::cat*), pUC-WXY63 | (Wang et al. 2021) |
| XJC01 | DH5α, pUC-P15A | This study |
| XJC02 | DH5α, pUC-P15A-NCDS | This study |
| XJC03 (XYC2017) | DH5α, pUC-WXY63 | (Wang et al. 2021) |
| XJC05 | DH10B, pUC-P15A | This study |
| XJC06 | DH10B, pUC-P15A-NCDS | This study |
| XJC07 (XYC8017) | DH10B, pUC-WXY63 | (Wang et al. 2021) |
| XJD2201-1 | BW25113 (Δ*ldhA, dld::cat*), pTrc99k-FDH3A3, pUC-WXY63 | This study |

**Supplementary Table** **S1.** Strains and plasmids used in this study (continued).

| Strains and plasmids | Characteristic | Source |
| --- | --- | --- |
| BL21(DE3) pTrc99K-FDH3A3 | Expression of formate dehydrogenase mutant 3A3 in BL21(DE3) | (Guo et al. 2020) |
| BL21(DE3) pTrc99K-FDH3C4 | Expression of formate dehydrogenase mutant 3C4 in BL21(DE3) | (Guo et al. 2020) |
| JW2892 | *E.coli* AG1, pCA24N-FbaA | (Kitagawa et al. 2005) |
| Sm-GAPN | BL21(DE3), pET28a-SmGAPN | This study |
| **Plasmids** |  |  |
| pUC-P15A | pUC18 with pBR322 ori replaced by P15A ori | (Wang et al. 2021) |
| pUC-P15A-NCDS | Express NCD synthase NcdS, pUC-P15A with *lacZ* replaced by *ara* operon | This study |
| pUC-WXY63 | Express NcdS, *Ft*NadE, *CtCTPS*,* pUC-P15A with *lacZ* replaced by *ara* operon | (Wang et al. 2021) |
| pk-2B3-1G6 | pK with lacZ substituted by phosphite dehydrogenase mutant Pdh_I151R/P176R/M207A | (Liu et al. 2019) |
| pTrc99k-FDH3A3 | pTrc99k with NCD preferred formate dehydrogenase mutant V198I/C256I/P260S/E261P/S381N/S383F | (Guo et al. 2020) |
| pCA24N-FbaA | pCA24N with fructose-bisphosphate aldolase class II (FbaA) from *E. coli* | (Kitagawa et al. 2005) |
| pET28a-SmGAPN | pET28a with glyceraldehyde-3-phosphate dehydrogenase (SmGAPN) from *Streptococcus mutans* | This study |

**Supplementary Table S2.** Amino acid sequences of overexpressed proteins.

| Name | Sequence | Description |
| --- | --- | --- |
| NcdS | MKSLQALFGGTFDPVHYGHLK**K**VETLANLIGLTRVTIIPNNVPPHRPQPEANSVQRKHMLELAIADKPLFTLDERELKRNAPS**V**TAQTLKEWRQEQGPDVPLAFIIGQDSLLTFPTW**D**EYETILDNAHLIV**L**RRPGYPLEMAQPQYQQWLEDHLTHNPEDLHLQPAGKIYLAETP**Y**FNISATIIRERLQNGESCEDLLPEPVLTYINQQGLYRHHHHHH | Mutant of NadD from *Escherichia coli* K-12 (UniProtKB/Swiss-Prot: P0A752.1) |
| *Ft*NadE | MKIVKDFSPKEYSQKLVNWLSDSCMNYPAEGFVIGLSGGIDSAVAASLAVKTGLPTTALILPSDNNQHQDMQDALELIEMLNIEHYTISIQPAYEAFLASTQSFTNLQNNRQLVIKGNAQARLRMMYLYAYAQQYNRIVIGTDNACEWYMGYFTKFGDGAADILPLVNLKKSQVFELGKYLDVPKNILDKAPSAGLWQGQTDEDEMGVTYQEIDDFLDGKQVSAKALERINFWHNRSHHKRKLALTPNFHHHHHH | From *Francisella tularensis* (GenBank: APS92582.1) |
| *Ct*CTPS* | MSFKSIFLTGGVVSSLGKGLTAASLALLLERQDLKVAMLKLDPYLNVDPGTMNPYEHGEVYVTDDGVETDLDLGHYHRFSSVQLSKYSIATSGQIYTKVLTKERNGEFLGSTVQVIPHVTNEIINVIQSCADHHKPDILIVEIGGTIG**E**IESLPFLEAVRQFRCEHPQDCLSIHMTYVPYLRAAKEIKTKPTQHSVQNLRSIGISPDVILCRSEAPLSTEVKRKISLFCNVPEHAVFNAIDLERSIYEMPLLLAKENISDFLLNKLGFSPKPLDLSDWQDLVEALCDKERQHVRIGLVGKYLEHKDAYKSVFEALFHASVPANCSLELVPIAPESEDLLEQLSQCDGCLIPGGFGTRSWEGKISAARYCREQNIPCFGICLGMQALVVEYARNVLDKPLANSMEMNPETPDPVVCMMEGQDSVVKGGTMRLGAYPCRIAPGSLASAAYKTDLVQERHRHRYEVNPSYIERLEEHGLKIAGVCPLGELCEIVEIPNHRWMLGVQFHPEFLSKLAKPHPLFIEFIRAAKAYSLEKANHEHRHHHHHH | Mutant of *Ct*CTPS from *Chlamydia trachomatis* (GenBank: AAA80195.1) |

Notes: Overexpressed proteins in NCD self-sufficient strains are NcdS, *Ft*NadE and *Ct*CTPS*. Mutated amino acids are in bold and underlined.

**Supplementary Table S3.** The genes deleted from *E. coli* K12 MG1655 to construct the host strain.

| Genotype | Protein | Gene | Abbreviation name in iML1515 | Number in iML1515 | GPR in iML1515 | Bound setting |
| --- | --- | --- | --- | --- | --- | --- |
| Δ*lacZ* | β-galactosidase | *lac*Z | LACZ | 294 | b0344 | 0 |
| Δ*araBAD* | ribulokinase | araB | RBK_L1 | 236 | b0063 | 0 |
|  | L-arabinose isomerase | *ara*A | ARAI | 472 | b0062 | 0 |
|  | L-ribulose-5-phosphate 4-epimerase | *ara*D | RBP4E | 237 | b0061 or b4198 or b3583 | unchanged |
| Δ*rhaBAD* | rhamnulokinase | *rha*B | RMK | 1402 | b3904 | 0 |
|  | L-rhamnose isomerase | *rha*A | RMI | 177 | b3903 | 0 |
|  | Lyxose isomerase |  | LYXI | 959 |  | 0 |
|  | rhamnulose-1-phosphate aldolase | *rha*D | RMPA | 328 | b3902 | 0 |
| Δ*ldhA* | D-lactate dehydrogenase | *ldh*A | LDH_D | 1693 | b1380 | 0 |
| *dld::cat* | D-lactate dehydrogenase | *dld* | LDH_D2 | 1639 | b2133 | 0 |

**Supplementary Table S4.** The reactions added to construct engineered strains *in silico*.

| Protein | Reaction | Lower bound | Upper bound |
| --- | --- | --- | --- |
| NCDS | NMN + CTP → NCD + ppi | 0 | 1000 |
| *Ct*CTPS* | UTP + ATP + glutamine → CTP + ADP + glutamate + pi | 0 | 1000 |
| *Ft*NadE | β-nicotinate D-ribonucleotide + ATP + glutamine → NMN + AMP + glutamate + pi | 0 | 1000 |

**Supplementary Table S5.** Specific growth rate of engineered strains in different media.

| Strain | Medium | Specific growth rate (μ_m_/h^-1^) | λ (h) |
| --- | --- | --- | --- |
| C1 | M9 | 0.46 ± 0.01 | 4.77 ± 0.09 |
|  | LB | 1.27 ± 0.02 | 0.69 ± 0.03 |
|  | TB | 1.63 ± 0.07 | 0.75 ± 0.08 |
| C2 | M9 | 0.46 ± 0.01 | 4.51 ± 0.14 |
|  | LB | 1.26 ± 0.03 | 0.51 ± 0.05 |
|  | TB | 1.62 ± 0.08 | 0.63 ± 0.10 |
| C3 | M9 | 0.45 ± 0.01 | 4.55 ± 0.14 |
|  | LB | 1.23 ± 0.03 | 0.40 ± 0.04 |
|  | TB | 1.58 ± 0.07 | 0.42 ± 0.08 |
| XJC01 | M9 | 0.23 ± 0.00 | 0.79 ± 0.08 |
|  | LB | 0.97 ± 0.02 | 1.30 ± 0.06 |
|  | TB | 1.02 ± 0.02 | 1.80 ± 0.06 |
| XJC02 | M9 | 0.2 ± 0.00 | 0.82 ± 0.08 |
|  | LB | 0.99 ± 0.02 | 1.15 ± 0.06 |
|  | TB | 1.02 ± 0.02 | 1.70 ± 0.06 |
| XJC03 | M9 | 0.25 ± 0.00 | 0.36 ± 0.08 |
|  | LB | 0.95 ± 0.01 | 1.07 ± 0.04 |
|  | TB | 1.03 ± 0.02 | 1.44 ± 0.06 |
| XJC05 | M9 | 0.82 ± 0.07 | 1.46 ± 0.10 |
|  | LB | 1.21 ± 0.02 | 0.29 ± 0.03 |
|  | TB | 1.20 ± 0.02 | 0.81 ± 0.05 |
| XJC06 | M9 | 0.79 ± 0.06 | 1.46 ± 0.10 |
|  | LB | 1.32 ± 0.03 | 0.20 ± 0.03 |
|  | TB | 1.00 ± 0.00 | 1.00 ± 0.00 |
| XJC07 | M9 | 0.76 ± 0.43 | 1.62 ± 0.12 |
|  | LB | 1.40 ± 0.02 | 0.28 ± 0.02 |
|  | TB | 1.24 ± 0.03 | 0.65 ± 0.05 |

**2. References**

Datsenko, K. A., Wanner, B. L. (2000). One-step inactivation of chromosomal genes in *Escherichia coli* K-12 using PCR products. *Proc. Natl. Acad. Sci. U. S. A.*  97(12), 6640-6645. doi:10.1073/pnas.120163297

Guo, X., Wang, X., Liu, Y., Li, Q., Wang, J., Liu, W., et al. (2020). Structure-guided design of formate dehydrogenase for regeneration of a non-natural redox cofactor. *Chem. Eur. J.* 26(70), 16611-16615. doi:10.1002/chem.202003102

Kitagawa, M., Ara, T., Arifuzzaman, M., Ioka-Nakamichi, T., Inamoto, E., Toyonaga, H., et al. (2005). Complete set of ORF clones of *Escherichia coli* ASKA library (a complete set of *E. coli* K-12 ORF archive): unique resources for biological research. *DNA Res.* 12(5), 291-299. doi:10.1093/dnares/dsi012

Liu, Y., Feng, Y., Wang, L., Guo, X., Liu, W., Li, Q., et al. (2019). Structural insights into phosphite dehydrogenase variants favoring a non-natural redox cofactor. *ACS Catal.* 9, 1883-1887. doi:10.1021/acscatal.8b04822

Wang, X., Feng, Y., Guo, X., Wang, Q., Ning, S., Li, Q., et al. (2021). Creating enzymes and self-sufficient cells for biosynthesis of the non-natural cofactor nicotinamide cytosine dinucleotide. *Nat. Commun.* 12(1), 2116. doi:10.1038/s41467-021-22357-z
